# Comparative estimation of the effects of antihypertensive medications on schizophrenia occurrence: a multinational observational cohort study

**DOI:** 10.1186/s12888-024-05578-6

**Published:** 2024-02-16

**Authors:** Dong Yun Lee, Chungsoo Kim, Jiwoo Kim, Jeongwon Yun, Yujin Lee, Celine Sze Ling Chui, Sang Joon Son, Rae Woong Park, Seng Chan You

**Affiliations:** 1 Department of Biomedical Informatics, Ajou University School of Medicine, 164, World cup-ro, Yeongtong-gu, Suwon-si, Gyeonggi-do 16499 Republic of Korea; 2 Department of Biomedical Sciences, Ajou University Graduate School of Medicine, Suwon, Korea; 3 Big Data Department, Health Insurance Review and Assessment Service, Wonju, Korea; 4 School of Nursing, Li Ka Shing Faculty of Medicine, The University of Hong Kong, Hong Kong Special Administration Region, Hong Kong, China; 5 School of Public Health, Li Ka Shing Faculty of Medicine, The University of Hong Kong, Hong Kong Special Administration Region, Hong Kong, China; 6 Laboratory of Data Discovery for Health (D24H), Hong Kong Science and Technology Park, Hong Kong Special Administration Region, Hong Kong Science Park, Hong Kong, China; 7 Department of Psychiatry, Ajou University School of Medicine, Suwon, Korea; 8 Department of Biomedicine Systems Informatics, Yonsei University College of Medicine, Seoul, Korea; 9 Institute for Innovation in Digital Healthcare, Yonsei University, 50-1 Yonsei-ro, Seodaemungu, Seoul, 03722 Republic of Korea

**Keywords:** Antihypertensive medications, Schizophrenia, Safety, Observational studies

## Abstract

**Background:**

The association between antihypertensive medication and schizophrenia has received increasing attention; however, evidence of the impact of antihypertensive medication on subsequent schizophrenia based on large-scale observational studies is limited. We aimed to compare the schizophrenia risk in large claims-based US and Korea cohort of patients with hypertension using angiotensin-converting enzyme (ACE) inhibitors versus those using angiotensin receptor blockers (ARBs) or thiazide diuretics.

**Methods:**

Adults aged 18 years who were newly diagnosed with hypertension and received ACE inhibitors, ARBs, or thiazide diuretics as first-line antihypertensive medications were included. The study population was sub-grouped based on age (> 45 years). The comparison groups were matched using a large-scale propensity score (PS)-matching algorithm. The primary endpoint was incidence of schizophrenia.

**Results:**

5,907,522; 2,923,423; and 1,971,549 patients used ACE inhibitors, ARBs, and thiazide diuretics, respectively. After PS matching, the risk of schizophrenia was not significantly different among the groups (ACE inhibitor vs. ARB: summary hazard ratio [HR] 1.15 [95% confidence interval, CI, 0.99–1.33]; ACE inhibitor vs. thiazide diuretics: summary HR 0.91 [95% CI, 0.78–1.07]). In the older subgroup, there was no significant difference between ACE inhibitors and thiazide diuretics (summary HR, 0.91 [95% CI, 0.71–1.16]). The risk for schizophrenia was significantly higher in the ACE inhibitor group than in the ARB group (summary HR, 1.23 [95% CI, 1.05–1.43]).

**Conclusions:**

The risk of schizophrenia was not significantly different between the ACE inhibitor vs. ARB and ACE inhibitor vs. thiazide diuretic groups. Further investigations are needed to determine the risk of schizophrenia associated with antihypertensive drugs, especially in people aged > 45 years.

**Supplementary Information:**

The online version contains supplementary material available at 10.1186/s12888-024-05578-6.

## Background

Schizophrenia is a mental disorder affecting approximately 1% of the world’s population and is a severe disorder that leads to functional deterioration [1]. Despite cardinal features of schizophrenia, it remains the least understood psychiatric disorder owing to the lack of pathological hallmarks [2, 3]. With the identification of schizophrenia susceptibility genes [4], genetic traits have been considered to play important roles in schizophrenia occurrence [5]. The relative contribution of genetic factors in schizophrenia is estimated to be up to 80% [6]. 

Recently, the target genes of antihypertensive medications were reported to be associated with the risk of schizophrenia. Specifically, low angiotensin-converting enzyme (ACE) messenger RNA and protein levels, which are targets of ACE inhibitors, are associated with an increased risk of schizophrenia [7]. In addition, according to Fan et al., genetically proxied ACE inhibitors were reported to be associated with an increased risk of SCZ in Europeans and East Asians [8]. Contrary to ACE inhibitors, other antihypertensive medications such as BB and CCB were found to have no association. Animal experiments demonstrated that the brain RAS targeted by ACE inhibitors can regulate various brain functions such as sensory information processing, learning, memory, and emotional responses [9]. However, it remains unclear whether this potential biological association translates into clinically significant difference of the schizophrenia occurrence in real-world scenarios. Given the widespread use of ACE inhibitors in hypertensive patients and their potential biological implications for schizophrenia risk, investigating this association using real-world data is essential.

Therefore, comparing the effects of antihypertensive drugs on schizophrenia may be a way to identify potential risk factors for schizophrenia occurrence. We aimed to conduct a head-to-head study comparing the occurrence of schizophrenia between antihypertensive drugs in patients with hypertension. Specifically, we investigated whether the use of ACE inhibitors increased the risk of schizophrenia compared with the use of angiotensin receptor blockers (ARBs) or thiazide diuretics in the US and Korea across the Observational Health Data Sciences and Informatics (OHDSI) network [10]. 

## Methods

### Data source

We performed a population-based, retrospective cohort study using two claims databases in the US and South Korea: US Open Claims and Health Insurance Review and Assessment Service National Claims (HIRA) (see eMethod 1 in Supplement 1 for database details). These databases were standardized using the Observational Medical Outcomes Partnership Common Data Model, version 5.3 [11]. 

Each data partner executes the package locally inside the firewall. The pre-designated statistical results (without patient-level information) were shared for interpretation and database-level meta-analyses. All partners received Institutional Review Board approval or exemption (IRB number: AJIRB-MED-MDB-21-274).

### Study design

Active-comparator new-user designs were applied in our study to mitigate the methodological limitations of observational studies [12]. For new-user design, we identified patients who had newly initiated antihypertensive medications. For the active-comparator design, ACE inhibitors were compared with ARBs and thiazide diuretics (thiazide or thiazide-like diuretics), which are commonly used for the same indication and reported to be unrelated to the occurrence of schizophrenia [13]. We compared the incidence of outcomes between the two groups (ACE inhibitor vs. ARB and ACE inhibitor vs. thiazide diuretics).

We conducted distributed network analyses similar to previous studies [14, 15]. The statistical analytical protocol (see Supplement 2) was pre-specified before execution. According to this protocol, the study package for the entire process was built using the OHDSI Health Analytics Data-to-Evidence Suite in R; detailed study codes are available online at https://github.com/ohdsi-studies/Ceeamos. The study protocol was registered with the EU Post-Authorization Studies register under EUPAS42783.

### Study population and exposure

We identified adult (aged ≥18 years) patients who were exposed to antihypertensive drugs (ACE inhibitors, ARBs, or thiazide diuretics) for the first time according to their medical history. Combination products of each ingredient were not included in this study. The index date was defined as the date of the first exposure to antihypertensive drugs. To avoid left censoring (i.e., incomplete data on patients who were already on antihypertensive treatment before entering the study), we excluded patients who were enrolled in the database for < 1 year before the index date. We excluded patients without a diagnosis 1 year before the index date. The other exclusion criteria were as follows: (1) a history of exposure to any hypertension treatment (prevalent user), (2) schizophrenia diagnosis and heart failure diagnosis at any time before the index date, (3) prescription of other blood pressure lowering medications (non-thiazide diuretics, beta blockers, and calcium channel blockers) and (4) prescription of the opposite drug (ARBs or thiazide diuretics for the ACE inhibitor group and vice versa) during the 7 days after the index date for ascertaining first-line treatment. Further details on cohort definitions are presented in Supplement 2.

### Outcomes and follow-up

The primary outcome was a diagnosis of schizophrenia for the first time. To increase the specificity of the diagnosis, we applied a restricted definition of outcome, which included at least one diagnosis of schizophrenia, at least two prescriptions of antipsychotics, or at least two psychiatric procedures (electroconvulsive therapy and psychotherapy) at any time after the first diagnosis of schizophrenia. The secondary outcome was a specific definition of schizophrenia at the emergency department visit. Further details of the outcome definitions are provided in Supplement 2.

Our analysis considered the time-to-first event and was followed up to the earliest date among last date of assigned treatment, date of last observation in the database, date of occurrence of the endpoint, and date of censoring (as-treated [AT] approach). Each treatment was considered to be continued if the patient received a new prescription for the same treatment within 30 days of the last date of the previous prescription. Treatment discontinuation was defined as the last prescription with no further prescription within 30 days. Censoring events were defined as events in which patients were no longer under the observation due to another antihypertensive medications. (i.e., patients in the ACE inhibitor group were considered censored if they were exposed to ARBs or thiazide diuretics).

### Statistical analysis

A large-scale propensity score (PS) adjustment [16] was performed using L1 penalized logistic regression, which used > 10 000 baseline patient characteristics between each of the two cohorts, including all available demographic characteristics, as well as the diagnosis, medication, and procedure history in each database. All variables were dichotomized, and missing variables were considered absent. The study populations were matched using variable-ratio PS matching with a maximum ratio of 10 (caliper = 0.2). Differences between the two matched cohorts were considered negligible when the absolute standardized mean differences (aSMDs) of all covariates were < 0.1 [17]. The incidence rates (IRs) per 1000 person-years (PY) were estimated. Cox proportional hazard models were used to estimate the association between exposure and outcomes. Next, we performed empirical calibration of all hazard ratio (HR) estimates, their 95% confidence intervals (CIs), and their 2-sided *P* values by fitting an empirical null distribution to point estimates of falsification end points [18]. We identified a total of 26 falsification endpoints to quantify systematic error (eTable 1 in Supplement 1) [19, 20]. These outcomes are not known to cause differences between antihypertensive drugs, such as ingrowing nails and fractures of the upper limb. Using calibrated estimates, we performed a random-effects meta-analysis to calculate the summary HR and 95% CI of the pooling effect estimates across the databases. The Kaplan–Meier method and log-rank tests were used to derive the cumulative incidence and comparative risk between-group differences. Statistical significance was set at a pre-specified two-sided *P* value < 0.05. We followed the Strengthening the Reporting of Observational Studies in Epidemiology (STROBE) reporting guidelines.

### Sensitivity analyses

Multiple sensitivity analyses were conducted using different definitions of the study population, outcomes, and follow-up strategies. To examine the association with late-onset schizophrenia as previously described [21], we sub-grouped the study population according to age over 45 years. We also varied our follow-up strategy to intention-to-treat (ITT) to estimate the effect of being assigned to a given treatment regardless of non-adherence. Overall, 16 different analyses (two cohort definitions (by age) × two outcome definitions × two follow-up strategies × two comparison pairs) were performed.

## Results

### Cohort characteristics

**Fig. 1:**
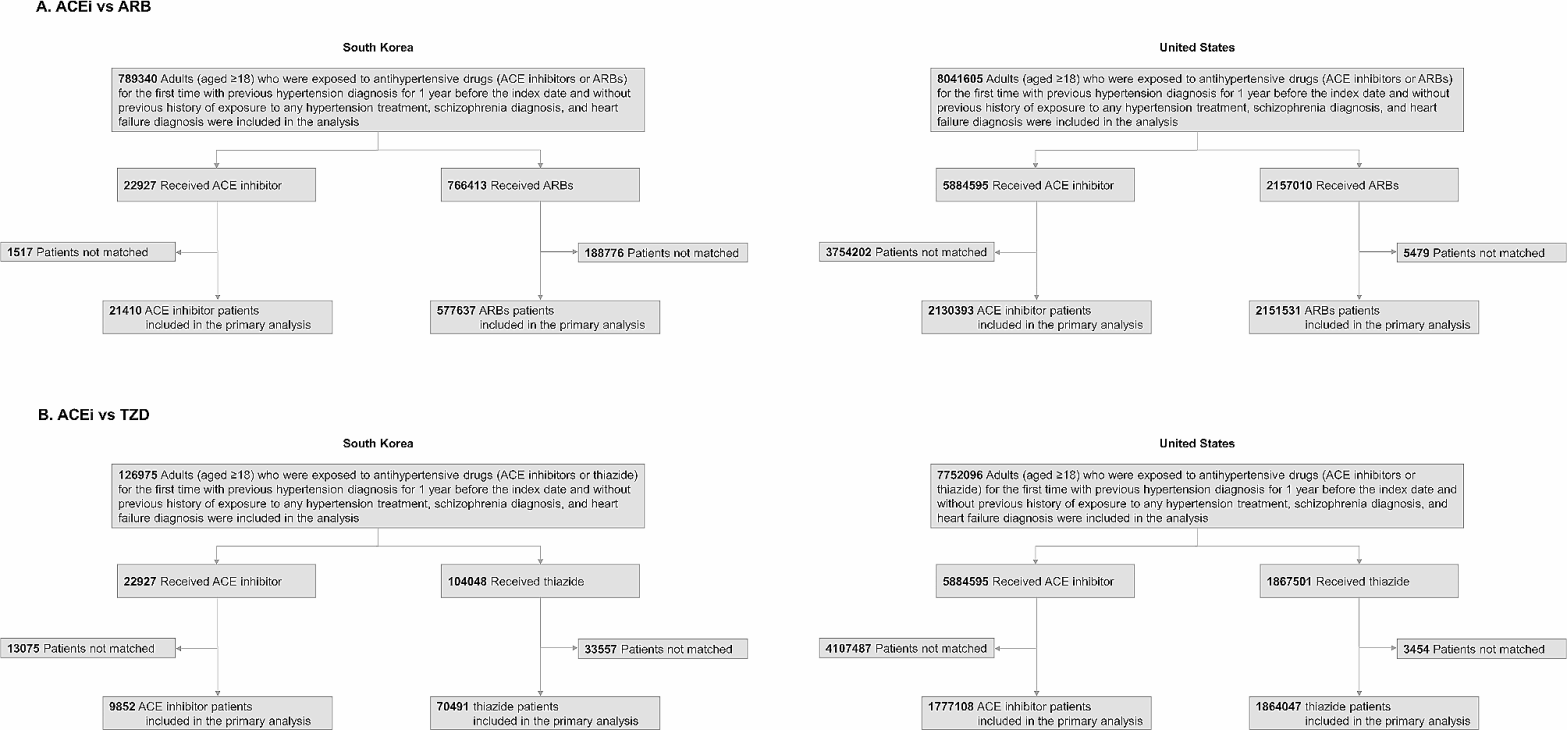
Flow diagram illustrating the identification of the study population in South Korea and the United States

In total, 5,907,522; 2,923,423; and 1,971,549 patients across the two databases for the three study populations (ACE inhibitor, ARB, and thiazide groups, respectively) were included in the analysis (Fig. 1). The number of matched patients in the ACE inhibitor versus ARB comparison was 21,410 and 577,637 pairs from the HIRA and 2,130,393 and 2,151,531 pairs from the US Open Claims database, respectively. In the ACE inhibitor versus thiazide diuretic comparison, there were 9,852 and 70,491 pairs from the HIRA and 1,777,108 and 1,864,047 pairs from the US Open Claims database, respectively. The baseline characteristics of the study populations before and after PS matching for the three target-comparator combinations are presented in eTable 2 (Supplement 1) and Table 1. After PS matching, the aSMD for all baseline patient characteristics between the two drug users was < 0.1 within each data source (eFigure 1 in Supplement 1).

**Table 1:** Comparisons of Baseline Characteristics Received ACE inhibitor, ARB, or Thiazide After Propensity Score Matching in South Korea and the United States

Patients Received ACE inhibitors or ARB							
	**No. (%)**						
	**South Korea**			**United States**			
**Characteristics**	**ACE inhibitor** **(** *n* ** = 21 410)**	**ARB** **(** *n* ** = 577 637)**	**aSMD**	**ACE inhibitor** **(** *n* ** = 2 130 393)**	**ARB** **(** *n* ** = 2 151 531)**	**aSMD**	
**Socio-demographics**							
Male	13 766 (64.3)	369 687 (64.0)	< 0.01	1 065 196 (50.0)	1 077 917 (50.1)	< 0.01	
< 45 years	8 607 (40.2)	232 210 (40.2)	< 0.01	752 029 (35.3)	761 642 (35.4)	< 0.01	
≥ 45 years	12 803 (59.8)	345 427 (59.8)	< 0.01	1 378 364 (64.7)	1 389 889 (64.6)	< 0.01	
**Medical history**							
Diabetes mellitus	6 508 (30.4)	186 576 (32.3)	0.04	430 339 (20.2)	436 760 (20.3)	< 0.01	
Hyperlipidemia	13 017 (60.8)	358 134 (62.0)	0.02	1 024 719 (48.1)	1 041 341 (48.4)	< 0.01	
Ischemic heart disease	3 511 (16.4)	91 266 (15.8)	0.02	61 781 (2.9)	60 242 (2.8)	< 0.01	
Atrial fibrillation	513 (2.4)	13 863 (2.4)	< 0.01	44 738 (2.1)	45 182 (2.1)	< 0.01	
Chronic kidney disease	471 (2.2)	14 440 (2.5)	0.02	70 302 (3.3)	73 152 (3.4)	< 0.01	
Cerebrovascular disease	1 370 (6.4)	39 279 (6.8)	0.01	85 215 (4.0)	86 061 (4.0)	< 0.01	
Depressive disorder	1 477 (6.9)	41 589 (7.2)	0.01	140 605 (6.6)	142 001 (6.6)	< 0.01	
Anxiety disorder	2 012 (9.4)	54 875 (9.5)	< 0.01	104 389 (4.9)	105 425 (4.9)	< 0.01	
**Medication use**							
Antidiabetics	7 900 (36.9)	228 744 (39.6)	0.06	317 428 (14.9)	318 426 (14.8)	< 0.01	
Lipid-lowering agents	9 056 (42.3)	250 116 (43.3)	0.02	594 379 (27.9)	602 428 (28.0)	< 0.01	
Anti-thrombotic agents	13 402 (62.6)	362 756 (62.8)	< 0.01	134 214 (6.3)	133 394 (6.2)	< 0.01	
Antidepressants	2 783 (13.0)	77 981 (13.5)	0.01	328 080 (15.4)	327 032 (15.2)	< 0.01	
Anxiolytics	5 630 (26.3)	151 918 (26.3)	< 0.01	189 605 (8.9)	189 334 (8.8)	< 0.01	
**Patients Received ACE inhibitors or Thiazide**							
	**No. (%)**						
	**South Korea**			**United States**			
**Characteristics**	**ACE inhibitor** **(** *n* **= 9 852)**	**Thiazide** **(** *n* **= 70 491)**	**aSMD**	**ACE inhibitor** **(** *n* **= 1 777 108)**	**Thiazide** **(** *n* **= 1 864 047)**	**aSMD**	
**Socio-demographics**							
Male	5 408 (54.9)	39 545 (56.1)	0.03	662 861 (37.3)	699 017 (37.5)	< 0.01	
< 45 years	3 566 (36.2)	25 024 (35.5)	0.01	630 873 (35.5)	680 377 (36.5)	0.04	
≥ 45 years	6 286 (63.8)	45 466 (64.5)	0.01	1 146 235 (64.5)	1 183 670 (63.5)	0.04	
**Medical history**							
Diabetes mellitus	2 157 (21.9)	17 058 (24.2)	0.05	188 373 (10.6)	193 860 (10.4)	< 0.01	
Hyperlipidemia	4 738 (48.1)	35 597 (50.5)	0.05	639 758 (36.0)	665 464 (35.7)	< 0.01	
Ischemic heart disease	886 (9.0)	6 837 (9.7)	0.03	31 987 (1.8)	33 552 (1.8)	< 0.01	
Atrial fibrillation	177 (1.8)	1 409 (2.0)	< 0.01	30 210 (1.7)	29 824 (1.6)	< 0.01	
Chronic kidney disease	98 (1.0)	775 (1.1)	< 0.01	35 542 (2.0)	35 416 (1.9)	0.01	
Cerebrovascular disease	610 (6.2)	4 581 (6.5)	0.02	53 313 (3.0)	52 193 (2.8)	0.01	
Depressive disorder	817 (8.3)	5 850 (8.3)	< 0.01	149 277 (8.4)	152 851 (8.2)	< 0.01	
Anxiety disorder	1 123 (11.4)	7 824 (11.1)	0.01	97 740 (5.5)	100 658 (5.4)	< 0.01	
**Medication use**							
Antidiabetics	2 226 (22.6)	17 834 (25.3)	0.06	108 403 (6.1)	111 842 (6.0)	< 0.01	
Lipid-lowering agents	2 699 (27.4)	20 583 (29.2)	0.04	300 331 (16.9)	316 888 (17.0)	< 0.01	
Anti-thrombotic agents	5 773 (58.6)	41 237 (58.5)	< 0.01	70 969 (4.5)	80 154 (4.3)	0.01	
Antidepressants	1 418 (14.4)	10 150 (14.4)	< 0.01	312 771 (17.6)	318 752 (17.1)	0.01	
Anxiolytics	3 014 (30.6)	20 865 (29.6)	0.02	181 265 (10.2)	186 404 (10.0)	0.01	

ACE, Angiotensin Converting Enzyme; ARB, Angiotensin II Receptor Blockers; PS: propensity score; aSMD: absolute standardized mean difference

### Primary outcome assessment

**Fig. 2:**
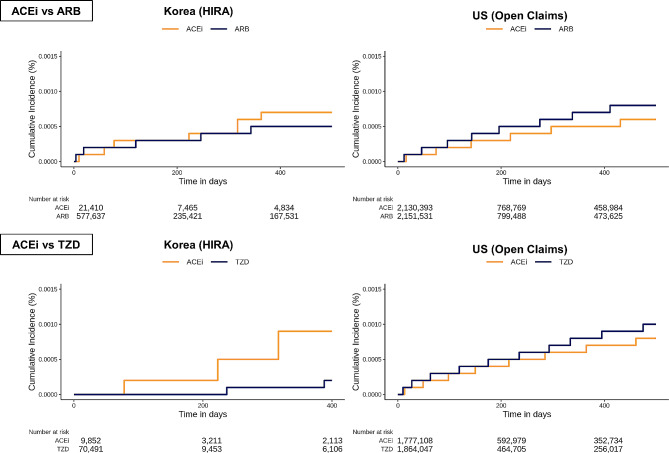
Kaplan–Meier plots for the risks of schizophrenia in propensity score-matched cohorts from each data source

**Fig. 3:**
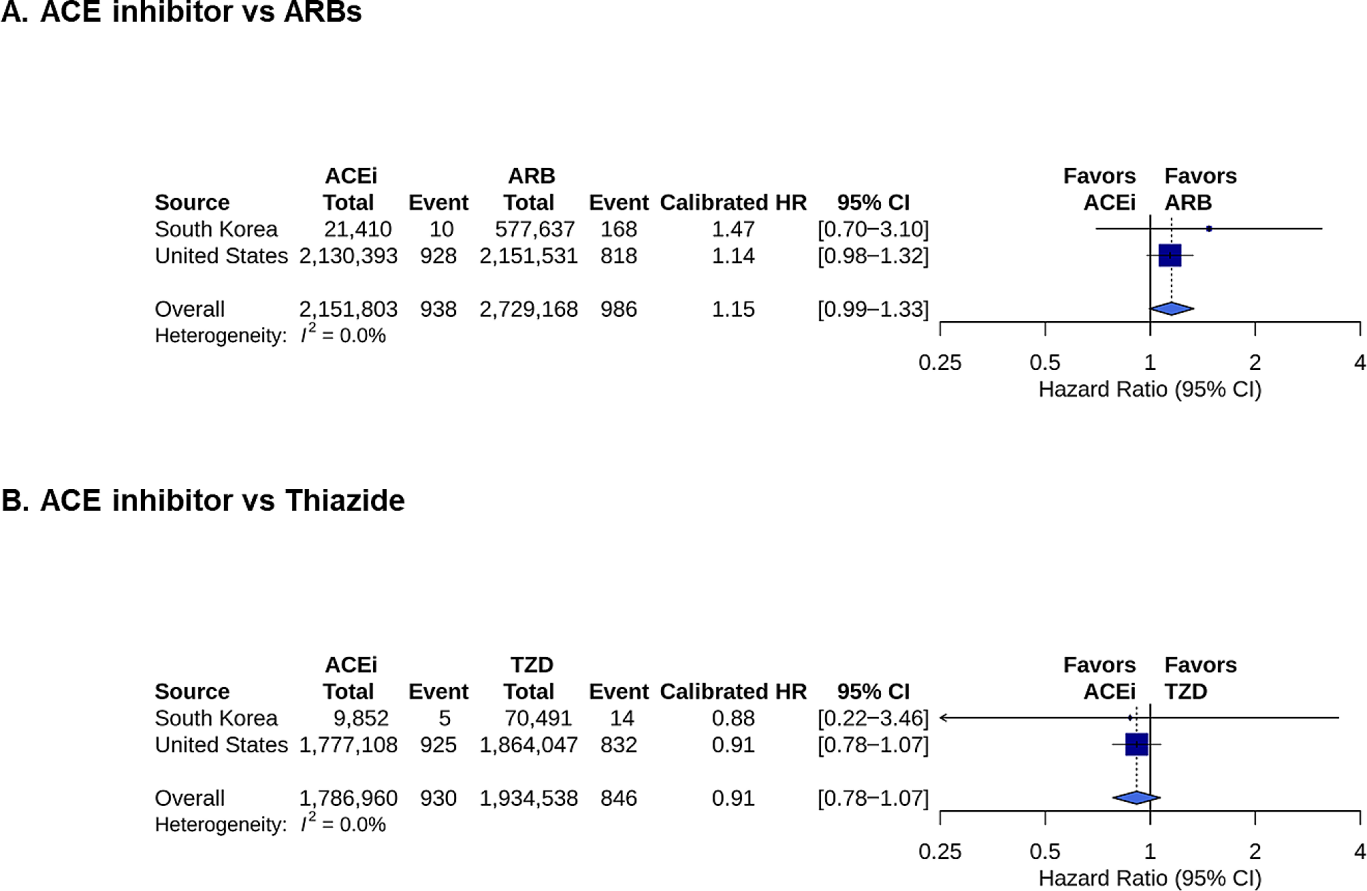
The forest plots for the risk of schizophrenia. Forest plots showing the calibrated HRs and 95% CIs for the occurrence of schizophrenia for each dataset. Summary HRs were calculated using a random-effects model. An HR of > 1 indicated a higher risk in the ACE inhibitor group. The size of the data marker indicates the weight of the study. Error bars represent 95% CIs. HR, hazard ratio; CI, confidence intervals; ACE, angiotensin-converting enzyme

The cumulative incidence curves for schizophrenia for all comparisons are shown in Fig. 2. Figure 3 shows the results of the meta-analyses, including the calibrated HR and 95% CI. The detailed numbers of events, PY, and IRs are presented in Table 2. In the US and South Korea, the comparison between the ACE inhibitor and ARB groups for the risk of schizophrenia occurrence was not significant (US: IR 0.43/1 000 PY, 0.37/1 000 PY, calibrated HR 1.14 [95% CI, 0.98–1.32]; Korea: IR 0.44/1 000 PY, 0.22/1 000 PY, HR 1.47 [95% CI, 0.70–3.10]) (Fig. 3**and** Table 2). Overall, the meta-analysis result showed no significant difference in schizophrenia occurrence between the ACE inhibitor and ARB groups (IR 0.43/1 000 PY, 0.33/1 000 PY, summary HR 1.15 [95% CI, 0.99–1.33], *P* =.06) (Fig. 3).

**Table 2:** Risk of outcome events between the ACE inhibitor versus the ARB groups or between the ACE inhibitor versus Thiazide groups

Outcomes	ACE inhibitor		ARB		Calibrated HR [95% CI]	ACE inhibitor		Thiazide		Calibrated HR [95% CI]
	N	IR^a^	N	IR^§^		N	IR^§^	N	IR^a^	
**South Korea**										
Total population	21 410	0.44	577 637	0.22	1.47 [0.70–3.10]	9 852	0.57	70 491	0.53	0.88 [0.22–3.46]
≥45 years	15 405	0.57	431 745	0.27	1.36 [0.64–2.88]	7 639	0.66	55 106	0.57	1.98 [0.42–9.21]
**United States**										
Total population	2 130 393	0.43	2 151 531	0.37	1.14 [0.98–1.32]	1 777 108	0.54	1 864 047	0.65	0.91 [0.78–1.07]
≥45 years	1 792 503	0.41	1 813 979	0.35	1.22 [1.04–1.43]^b^	1 316 424	0.47	1 359 260	0.54	0.89 [0.74–1.07]

^a^Incidence rates were calculated as case per 1 000 person-years; ^b^statistically significant

ACE, Angiotensin Converting Enzyme; ARB, Angiotensin II Receptor Blockers, IR: Incidence rates; HR: hazard ratio; CI: 95% confidence interval

In the ACE inhibitor group, compared with the thiazide group, the risk was not significantly different between the US and South Korea (US: IR 0.54/1 000 PY, 0.65/1 000 PY, calibrated HR 0.91 [95% CI, 0.78–1.07]; Korea: IR 0.57/1 000 PY, 0.53/1 000 PY, calibrated HR 0.88 [95% CI, 0.22–3.46]) (Fig. 3**and** Table 2). Additionally, the meta-analysis result showed no significant difference in schizophrenia occurrence between the ACE inhibitor and thiazide groups (IR 0.54/1 000 PY, 0.64/1 000 PY, summary HR 0.91 [95% CI, 0.78–1.07], *P* =.26) (Fig. 3).

### Secondary outcome assessments

The meta-analysis of secondary outcomes is shown in eFigure 3 (Supplement 1). There was no significant difference in schizophrenia occurrence between the ACE inhibitor and ARB groups (summary HR 1.19 [95% CI, 0.59–2.39], *P* =.62). Similarly, no significant difference in schizophrenia occurrence was observed between the ACE inhibitor and thiazide groups (summary HR 0.87 [95% CI, 0.54–1.42]; *P* =.59).

### Falsification endpoint analyses and sensitivity analyses

In the analyses of falsification endpoints between the ACE inhibitor and ARB groups, 95.5% (21/22) in South Korea and 80.8% (21/26) in the United States had 95% CIs that covered 1.0 of the HR, suggesting that the level of systematic error was modest (eFigure 2 in Supplement 1). In another comparison between the ACE inhibitor and thiazide groups, the level of systematic error after calibration was also modest (South Korea: 77.8% [14/18]; United States: 73.1% [19/26] of the nominal 95% CIs covered 1.0) (eFigure 2 in Supplement 1).

In the subgroup analyses regarding age over 45 years to identify the relationship with late-onset schizophrenia, there was no significant difference in the schizophrenia occurrence between the ACE inhibitor and thiazide groups (summary HR 0.91 [95% CI, 0.71–1.16]; *P* =.44). However, the ACE inhibitor group showed a higher risk of schizophrenia occurrence than the ARB group (summary HR 1.23 [95% CI, 1.05–1.43]; *P* =.01, see eFigure 4 in Supplement 1). For the secondary outcome in the subgroups, there was no significant difference in the occurrence of schizophrenia in all comparisons between the target and comparator groups (eFigure 5 in Supplement 1).

The results of the additional follow-up strategy are presented in eTable 3 and eFigures 6–9 in Supplement 1. For the primary outcome at ITT follow-up, the ACE inhibitor group showed a lower risk of schizophrenia occurrence than the thiazide group (summary HR 0.92 [95% CI, 0.86–0.99]; *P* =.02). However, no significant difference was observed in schizophrenia occurrence in the meta-analysis for other comparisons of the target and comparator groups, including the total population and subgroups. In the US results, the ACE inhibitor group showed a higher risk of schizophrenia occurrence than the ARB group, including the total population and the subgroup (total population: calibrated HR 1.21 [95% CI, 1.13–1.29]; subgroup: calibrated HR 1.18 [95% CI, 1.09–1.27]). The ACE inhibitor group showed a lower risk of schizophrenia occurrence than the thiazide group in only the total population (total population: calibrated HR 0.91 [95% CI, 0.85–0.98]; subgroup: calibrated HR 0.96 [95% CI, 0.88–1.04]). Conversely, the results from South Korea showed no significant difference in the occurrence of schizophrenia for all comparisons between the target and comparator. eFigure 6 and eFigure 7 in Supplement 1). For the secondary outcome at ITT follow-up, the ACE inhibitor group exhibited a higher risk of schizophrenia occurrence than the ARB group (summary HR 1.27 [95% CI, 1.07–1.51]; *P* =.006). However, there was no significant difference in the occurrence of schizophrenia in the meta-analysis for other comparisons of the target and comparator groups, including the total population and subgroups. In the US results, the ACE inhibitor group showed a higher risk of schizophrenia occurrence than the ARB group, including the total population and the subgroup (total population: calibrated HR 1.29 [95% CI, 1.08–1.54]; subgroup: calibrated HR 1.24 [95% CI, 1.01–1.52]). However, the ACE inhibitor group showed a lower risk of schizophrenia occurrence than the thiazide group in only the total population (total population: calibrated HR 0.86 [95% CI, 0.75–0.99]; subgroup: calibrated HR 0.88 [95% CI, 0.73–1.06]). Contrary to the results from the US, those from South Korea showed no significant difference in the occurrence of schizophrenia for all comparisons between the target and comparator. (eFigure 8 and eFigure 9 in Supplement 1).

## Discussion

The potential association between ACE inhibitors and an increased risk of schizophrenia is highly relevant because of the number of affected patients and the burden of schizophrenia, warranting thorough investigation. In this study, we extensively estimated the comparative risks of ACE inhibitors and thiazide diuretics or ARBs on the occurrence of schizophrenia. No differences in risk were found between the use of ACE inhibitors versus ARB or between the use of ACE inhibitors versus thiazide diuretics. Although the use of ACE inhibitors was associated with an increased risk of schizophrenia compared with the use of ARB in the group aged > 45 years, the results were not consistent in the sensitivity analyses. Regarding the secondary outcome, no difference in risk was found among the antihypertensive drugs.

Schizophrenia imposes significant health, social, and economic burdens on individuals, families, caregivers, and society at large [22]. Unfortunately, by the time schizophrenia becomes apparent behaviorally, neural damages may already be irreversible [23]. Owing to the limited effectiveness of treatments, identifying psychosis risk factors for prevention and early detection has become crucial [24]. Additionally, regarding antihypertensive medications, 31.1% of adults worldwide are affected by hypertension [25]. ACE inhibitors are the most commonly used antihypertensive medications in the US. Previous studies on the relationship between ACE inhibitors and schizophrenia have limitations in terms of sample size or cross-sectional design [26, 27]. Therefore, we conducted this well-designed longitudinal cohort study. First, we selected the fit-for-purpose databases for the two countries. The large claims database has less fragmentation than individual electronic medical records, allowing us to conduct longitudinal cohort studies for identifying genetic relationships in schizophrenia [28]. Given the different prevalence of schizophrenia between countries [29], we analyzed more than 2 million patients in the United States and South Korea. In particular, the HIRA database contains nationwide claims data for the entire Korean population; therefore, our results are sufficiently representative. Second, many robust designs and methods were applied to infer associations between study groups. Controlling biases is critical in observational studies using routinely collected observational databases [30]. Using an active-comparator new-user design, large-scale PS methods can resolve biases arising from time-related design and comparability [31, 32]. An assessment of systematic errors using falsification endpoints also provides a more reliable statistical interpretation and minimizes the effect of residual bias [18]. 

Additionally, in the subgroup of individuals aged ≥ 45 years, a significant difference was observed between ACE inhibitors and ARBs. The possible biological pathway for the association between ACE inhibitors and schizophrenia is that ACE and the central RAS may play a role in inflammation and immunity [33]. Immune dysfunction due to reduced ACE activity may contribute to the development of schizophrenia [34]. Especially, according to studies on the pharmacokinetics of ACE inhibitors, the Area Under the Plasma Concentration-Time Curve has been reported to be greater in older individuals compared to younger ones, attributed to renal function decline and changes in body composition [35]. These findings suggest that the impact of ACE inhibitors is more pronounced in older individuals, as in our results. As another possible explanation, a previous study suggested the therapeutic potential of ARBs in patients with schizophrenia through the anti-inflammatory properties of gamma-aminobutyric acid [36], while thiazide diuretics had no effect on schizophrenia [13]. This could explain why ARBs are associated with a lower risk than ACE inhibitors. Nonetheless, the results were not significant in the ITT setting and require further study, making it difficult to draw definitive conclusions.

Moreover, differences in the risk of schizophrenia based on antihypertensive medication existed in the US data at the ITT follow-up. Although the differences between ACE inhibitors and thiazide diuretics were inconsistent, ACE inhibitors were consistently associated with a higher risk of schizophrenia than ARBs in both subgroup analyses and secondary outcomes. This result appears to be consistent with the results of the subgroup analysis at the AT follow-up. However, ITT can overestimate the effects of treatment in the presence of differential adherence [37]. 

Hypertensive patients are more likely to be diagnosed with mental disorders, and hypertension increases the severity of psychological distress. On the contrary, mental disorders are independent risk factors for hypertension. In other words, there is a clinically significant bidirectional relationship between hypertension and mental disorders [38–40]. In these situations, it is important to clarify how and to what extent antihypertensives affect schizophrenia from a clinical perspective. Given our findings of no significant differences by antihypertensive medication, there is insufficient evidence to recommend clinically that antihypertensive medications be reduced or discontinued. From the patient’s perspective, information about hypertension medications associated with schizophrenia risk could impact treatment adherence for people with hypertension, given what has happened to them during COVID-19 [41]. Considering our results, it does not appear that people with hypertension need to consider whether to use or change their antihypertensive medication because of the risk of schizophrenia.

This study had some limitations. First, there may be unmeasured risk factors for schizophrenia. For example, the balance for hypertension status (including blood pressure values) between the two groups could not be determined due to the nature of the claims data. A family history of schizophrenia and social history, such as immigration, are related to the development of schizophrenia [42, 43]. In addition, economic variables (such as income status) may also be associated with the development of schizophrenia, but were not used in this study. However, we used large-scale PS methods that can help reduce the impact of measured confounders and balance the distribution of these variables between groups [44]. Nevertheless, given the uncontrolled confounding by the propensity score method, further studies including social and family factors are needed. Second, the number of patients varies across the databases. Although data from 50 million people in Korea were used, only approximately 22,927 patients used ACE inhibitors, while data from the United States exceeded 5 million patients using ACE inhibitors. Such discrepancies in sample sizes could potentially impact the generalizability of our findings [45]. However, it is important to note that this heterogeneity in clinical practice can also be seen as a strength of our study. By utilizing data with diverse prescribing patterns, we can generate more reliable and generalizable evidence that better reflects real-world clinical scenarios. Moreover, the use of multinational databases and studies with multiple databases of varying sizes have previously demonstrated feasible and consistent results [46]. Third, the diagnostic system for schizophrenia has limitations. Prior reviews have shown variability in schizophrenia diagnosis [47], which may be due to the complexity and heterogeneity of schizophrenia [48]. These diagnostic problems appear not only in schizophrenia but also in other psychiatric diseases such as depression and bipolar disorder [49]. For the strictness of diagnosis, we added prescriptions of antipsychotics and occurrences of psychiatry procedures. Lastly, more comprehensive analyses are still needed to generalize our findings. This study only included RAS inhibitors and thiazide diuretics among the main antihypertensive drugs, and additional analyses such as calcium channel blockers could be considered. It also excluded patients on two or more medications, which are prescribed to more than half of all patients with hypertension [50], and further research is needed on patients on such combination therapies.

## Conclusions

In conclusion, there was no explicit difference in the risk of schizophrenia between ACE inhibitors, ARBs, and thiazide diuretics across the two large databases in the US and South Korea. These results are not sufficient to justify a change in current prescribing guidelines in hypertensive patients because of the risk of schizophrenia. Considering the unmeasured confounders, further investigations are needed to clarify the association between schizophrenia and antihypertensive drugs.

### Electronic supplementary material

Below is the link to the electronic supplementary material.

Supplementary Material 1

Supplementary Material 2

Supplementary Material 3

## Data Availability

Data are available from the corresponding authors upon reasonable request and with permission of HIRA and IQVIA.
