# Supplementary material for: Comparative estimation of the effects of antihypertensive medications on schizophrenia occurrence: a multinational observational cohort study
Source: BMC Psychiatry. 2024 Feb 16;24:128. doi: 10.1186/s12888-024-05578-6 (PMC10870661; doi:10.1186/s12888-024-05578-6)

**Supplement 1**

Supplement to “Comparative Estimation of the Effects of Anti-hypertensive Medications on the Occurrence of Schizophrenia”

eMethods 1. Description of data sources

**Health Insurance Review and Assessment Service National Claims (South Korea HIRA)**

*Data used in this study was updated to 2023/04*

The HIRA database covers a time period from 2010 to 2020. The database contains complete health information for the Korean population (50M population), including anonymized personal identifiers, demographics, diagnoses, and information on medical procedures and medications in the national reimbursement lists. The HIRA database was standardized to Observational Medical Outcomes Partnership common data model (OMOP–CDM) version 5.3.

**US Open Claims**

*Data used in this study was updated to 2021/10*

US Open Claims comprises pre-adjudicated professional and medical claims at the anonymized patient level collected from office management software and clearinghouse switch sources for the purpose of reimbursement. It includes adjudicated claims for a subset of medical claims data. The database covers a time period from 2000 to the present.11.7M providers contribute to the database covering 150M active patients (~50% of the US national population). Observation time is defined by the first and last consultation dates. The database was standardized to Observational Medical Outcomes Partnership common data model (OMOP–CDM) version 5.3.

eTable 1. The list of falsification endpoints

| **OMOP Concept ID** | **SNOMED code** | **Outcome Name** |
| --- | --- | --- |
| 4088290 | 248802009 | Absence of breast |
| 4092879 | 249582007 | Absent kidney |
| 432595 | 17602002 | Amyloidosis |
| 77650 | 398199007 | Aseptic necrosis of bone |
| 133655 | 80827001 | Burn of forearm |
| 73560 | 55260003 | Calcaneal spur |
| 140842 | 274672009 | Changes in skin texture |
| 78619 | 22878006 | Contusion of knee |
| 433527 | 129103003 | Endometriosis |
| 73008 | 23680005 | Enthesopathy |
| 433111 | 212966005 | Effects of hunger |
| 4345472 | 240530001 | Epstein-Barr virus disease |
| 4050747 | 23406007 | Fracture of upper limb |
| 433577 | 122481008 | Hammer toe |
| 441818 | 400210000 | Hemangioma |
| 4231770 | 439698008 | Hereditary thrombophilia |
| 4038835 | 118599009 | Hodgkin's disease |
| 380688 | 267384006 | Hypoglycemic coma |
| 4344500 | 239960007 | Impingement syndrome of shoulder region |
| 139099 | 400097005 | Ingrowing nail |
| 444132 | 125601008 | Injury of knee |
| 434203 | 80350001 | Late effect of contusion |
| 4215978 | 414941008 | Onychomycosis |
| 4202045 | 51771007 | Postviral fatigue syndrome |
| 134461 | 30128009 | Tietze's disease |
| 140641 | 57019003 | Verruca vulgaris |

eTable 2. Comparisons of Baseline Characteristics Received ACE inhibitor, ARB, or Thiazide before propensity score matching in South Korea and United States

| **Patients** **Received ACE inhibitor or ARB** | | | | | | |
| --- | --- | --- | --- | --- | --- | --- |
|  | **No. (%)** |  |  |  |  |  |
|  | **South Korea** | | | **United States** | | |
| **Characteristics** | **ACE inhibitor (n=21 545)** | **ARB (n=759 971)** | **aSMD** | **ACE inhibitor (n=5 871 325)** | **ARB (n=2 151 532)** | **aSMD** |
| **Socio-demographics** | | | | | | |
| Male | 13 724 (63.7) | 458 262 (60.3) | 0.07 | 3 229 229 (55.0) | 1 073 614 (49.9) | 0.10 |
| < 45 years | 8 489(39.4) | 301 708 (39.7) | 0.07 | 2 518 798 (42.9) | 755 187 (35.1) | 0.30 |
| ≥45 years | 13 056 (60.6) | 458 263 (60.3) | 0.02 | 3 352 527 (57.1) | 1 396 344 (64.9) | 0.31 |
| **Medical history** | | | | | | |
| Diabetes mellitus | 6 377 (29.6) | 154 274 (20.3) | 0.22 | 1 332 791 (22.7) | 438 912 (20.4) | 0.06 |
| Hyperlipidemia | 12 776 (59.3) | 346 546 (45.6) | 0.28 | 2 595 126 (44.2) | 1 045 645 (48.6) | 0.09 |
| Ischemic heart  disease | 3 576 (16.6) | 29 638 (3.9) | 0.43 | 158 525 (2.7) | 60 242 (2.8) | 0.01 |
| Atrial fibrillation | 495 (2.3) | 3 039 (0.4) | 0.17 | 99 812 (1.7) | 45 182 (2.1) | 0.03 |
| Chronic kidney  disease | 452 (2.1) | 7 599 (1.0) | 0.09 | 140 911 (2.4) | 75 303 (3.5) | 0.06 |
| Cerebrovascular  disease | 1 357 (6.3) | 30 398 (4.0) | 0.11 | 228 981 (3.9) | 86 061 (4.0) | 0.01 |
| Depressive disorder | 1 486 (6.9) | 38 758 (5.1) | 0.08 | 446 220 (7.6) | 142 001 (6.6) | 0.04 |
| Anxiety disorder | 2 025 (9.4) | 60 797 (8.0) | 0.05 | 293 566 (5.0) | 105 425 (4.9) | 0.01 |
| **Medication use** | | | | | | |
| Antidiabetics | 7 648 (35.5) | 151 994 (20.0) | 0.35 | 1 121 423 (19.1) | 316 275 (14.7) | 0.12 |
| Lipid-lowering agents | 8 854 (41.1) | 185 432 (24.4) | 0.36 | 1 667 456 (28.4) | 604 580 (28.1) | 0.01 |
| Anti-thrombotic  agents | 13 465 (62.5) | 387 585 (51.0) | 0.23 | 387 507 (6.6) | 135 546 (6.3) | 0.01 |
| Antidepressants | 2 822 (13.1) | 74 477 (9.8) | 0.11 | 986 382 (16.8) | 327 032 (15.2) | 0.04 |
| Anxiolytics | 5 752 (26.7) | 182 393 (24.0) | 0.06 | 551 904 (9.4) | 189 334 (8.8) | 0.02 |
|  | | | | | | |

eTable 2. Comparisons of Baseline Characteristics Received ACE inhibitor, ARB, or Thiazide before propensity score matching in South Korea and United States (Continued)

| **Patients Received ACE inhibitor or Thiazide** | | | | | | |
| --- | --- | --- | --- | --- | --- | --- |
|  | **No. (%)** |  |  |  |  |  |
|  | **South Korea** | | | **United States** | | |
| **Characteristics** | **ACE inhibitor (n=21 545)** | **Thiazide (n=98 355)** | **aSMD** | **ACE inhibitor (n=5 871 325)** | **Thiazide (n=1 864 056)** | **aSMD** |
| **Socio-demographics** | | | | | | |
| Male | 13 724 (63.7) | 32 457 (33.0) | 0.64 | 3 229 229 (55.0) | 676 652 (36.3) | 0.38 |
| < 45 years | 8 489(39.4) | 37 080 (37.7) | 0.02 | 2 518 798 (42.9) | 691 565 (37.1) | 0.15 |
| ≥45 years | 13 056 (60.6) | 61 275 (62.3) | 0.02 | 3 352 527 (57.1) | 1 172 491 (62.9) | 0.16 |
| **Medical history** | | | | | | |
| Diabetes mellitus | 6 377 (29.6) | 12 786 (13.0) | 0.41 | 1 332 791 (22.7) | 188 269 (10.1) | 0.35 |
| Hyperlipidemia | 12 776 (59.3) | 35 407 (36.0) | 0.48 | 2 595 126 (44.2) | 656 147 (35.2) | 0.18 |
| Ischemic heart  disease | 3 576 (16.6) | 4 622 (4.7) | 0.39 | 158 525 (2.7) | 33 553 (1.8) | 0.06 |
| Atrial fibrillation | 495 (2.3) | 4 917 (0.5) | 0.16 | 99 812 (1.7) | 29 824 (1.6) | <0.01 |
| Chronic kidney  disease | 452 (2.1) | 3 934 (0.4) | 0.15 | 140 911 (2.4) | 35 417 (1.9) | 0.04 |
| Cerebrovascular  disease | 1 357 (6.3) | 7 081 (7.2) | 0.04 | 228 981 (3.9) | 52 193 (2.8) | 0.06 |
| Depressive disorder | 1 486 (6.9) | 11 015 (11.2) | 0.15 | 446 220 (7.6) | 154 716 (8.3) | 0.03 |
| Anxiety disorder | 2 025 (9.4) | 18 294 (18.6) | 0.27 | 293 566 (5.0) | 102 523 (5.5) | 0.02 |
| **Medication use** | | | | | | |
| Antidiabetics | 7 648 (35.5) | 8 655 (8.8) | 0.68 | 1 121 423 (19.1) | 108 115 (5.8) | 0.41 |
| Lipid-lowering  agents | 8 854 (41.1) | 14 359 (14.6) | 0.62 | 1 667 456 (28.4) | 309 433 (16.6) | 0.28 |
| Anti-thrombotic  agents | 13 465 (62.5) | 58 914 (59.9) | 0.05 | 387 507 (6.6) | 82 018 (4.4) | 0.10 |
| Antidepressants | 2 822 (13.1) | 16 917 (17.2) | 0.11 | 986 382 (16.8) | 322 481 (17.3) | 0.01 |
| Anxiolytics | 5 752 (26.7) | 43 866 (44.6) | 0.38 | 551 904 (9.4) | 190 133 (10.2) | 0.03 |
| ACE, Angiotensin Converting Enzyme; ARB, Angiotensin II Receptor Blockers; PS: propensity score; aSMD: absolute standardized mean difference. | | | | | | |

eFigure 1. Scatter plots between the ACE inhibitor and the ARB group or between the ACE inhibitor and Thiazide groups before and after the propensity score adjustment

**ACE inhibitor vs ARB**


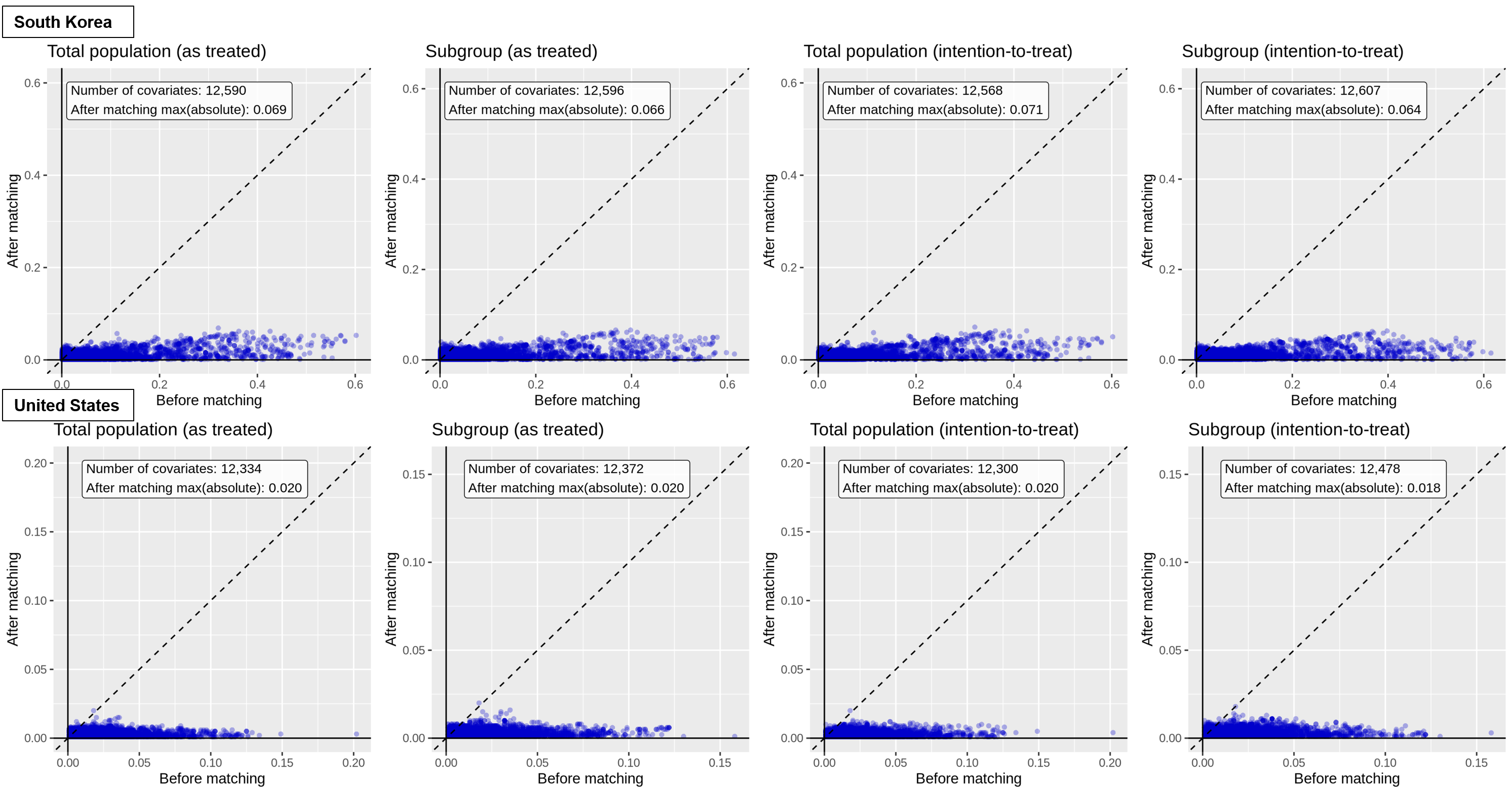


**ACE inhibitor vs thiazide**


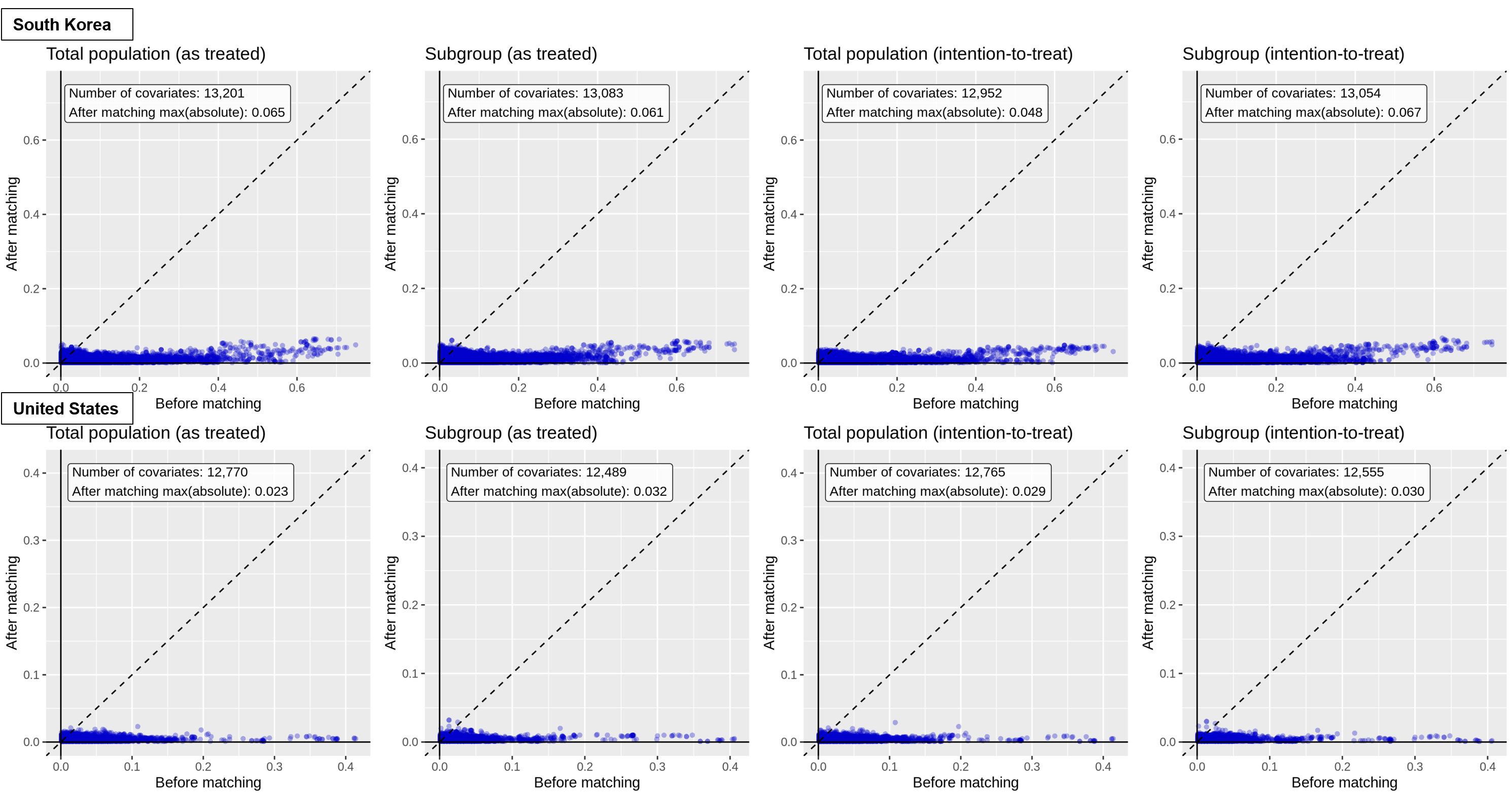


**eFigure 2. Systematic error control of falsification endpoints between the ACE inhibitor and the ARB group or between the ACE inhibitor and Thiazide groups under on-treatment, variable ratio propensity score matching design**

**ACE inhibitor vs ARB**


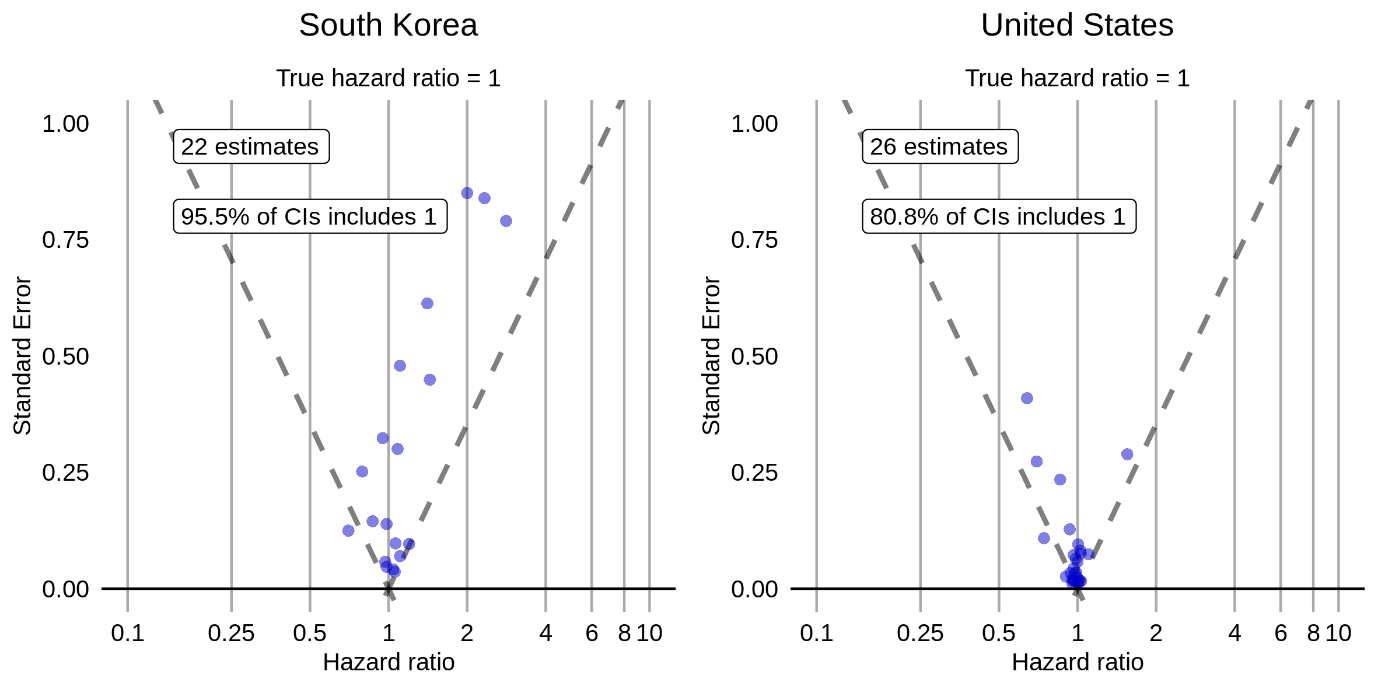


**ACE inhibitor vs thiazide**


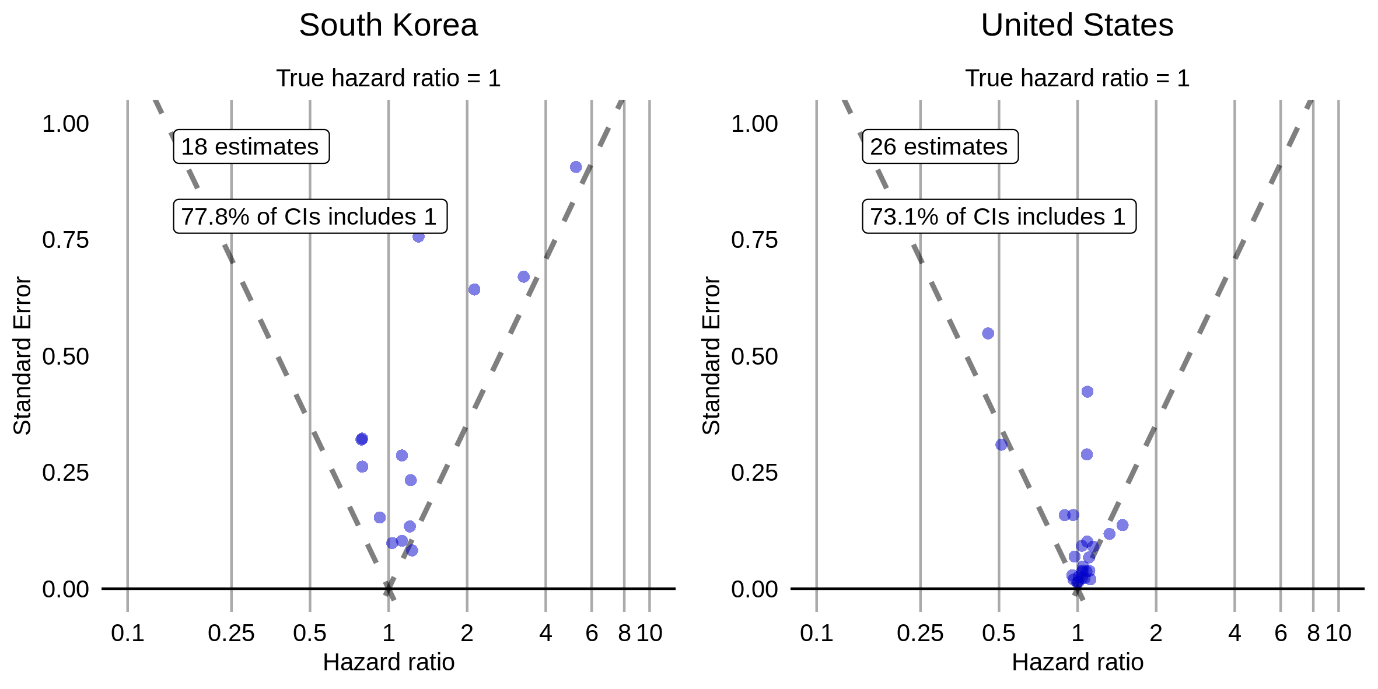


eTable 3. Risk of outcomes between the ACE inhibitor and the ARB group or between the ACE inhibitor and Thiazide groups across various statistical approaches

| **Outcomes** | **ACE inhibitor** | | **ARB** | | **Calibrated HR**  **[95% CI]** | **ACE inhibitor** | | **Thiazide** | | **Calibrated HR**  **[95% CI]** |
| --- | --- | --- | --- | --- | --- | --- | --- | --- | --- | --- |
|  | **Events** | **IR**^§^ | **Events** | **IR**^§^ |  | **Events** | **IR**^§^ | **Events** | **IR**^§^ |  |
| **South Korea** | | | | | | | | | | |
| **Primary outcome (schizophrenia)** | | | | | | | | | | |
| **Total patients** |  |  |  |  |  |  |  |  |  |  |
| As treated | 21 410 | 0.44 | 577 637 | 0.22 | 1.51 [0.69-3.03] | 9 852 | 0.57 | 70 491 | 0.53 | 0.97 [0.24-3.71] |
| Intention-to-treat | 22 759 | 0.34 | 602 366 | 0.29 | 0.81 [0.59-1.07] | 11 127 | 0.40 | 77 549 | 0.46 | 1.08 [0.67-1.67] |
| **≥45 years** |  |  |  |  |  |  |  |  |  |  |
| As treated | 15 405 | 0.57 | 431 745 | 0.27 | 1.35 [0.61-2.73] | 7 639 | 0.66 | 55 106 | 0.57 | 2.25 [0.49-10.59] |
| Intention-to-treat | 16 487 | 0.41 | 453 994 | 0.36 | 0.75 [0.54-1.02] | 8 663 | 0.50 | 60 596 | 0.56 | 1.27 [0.78-2.00] |
| **Secondary outcome (schizophrenia with ER visit)** | | | | | | | | | | |
| **Total patients** |  |  |  |  |  |  |  |  |  |  |
| As treated | 21 410 | 0.04 | 577 637 | 0.02 | 1.53 [0.13-18.06] | 9 852 | 0.11 | 70 491 | 0.08 | 5.39 [0.12-248.18] |
| Intention-to-treat | 22 759 | 0.04 | 602 366 | 0.02 | 0.90 [0.37-2.71] | 11 127 | 0.05 | 77 549 | 0.04 | 2.96 [0.81-10.81] |
| **≥45 years** |  |  |  |  |  |  |  |  |  |  |
| As treated | 15 405 | 0.06 | 431 745 | 0.03 | 0.89 [0.08-9.71] | 7 639 | 0.13 | 55 106 | 0.09 | 10.10 [0.29-353.15] |
| Intention-to-treat | 16 487 | 0.05 | 453 994 | 0.03 | 0.91[0.35-2.40] | 8 663 | 0.05 | 60 596 | 0.05 | 1.43 [0.33-6.14] |
| **(Continue)** | | | | | | | | | | |

eTable 3. Risk of outcomes between the ACE inhibitor and the ARB group or between the ACE inhibitor and Thiazide groups across various statistical approaches (continued)

| **Outcomes** | **ACE inhibitor** |  | **ARB** |  | **Calibrated HR**  **[95% CI]** | **ACE inhibitor** |  | **Thiazide** |  | **Calibrated HR**  **[95% CI]** |
| --- | --- | --- | --- | --- | --- | --- | --- | --- | --- | --- |
|  | **Events** | **IR**^§^ | **Events** | **IR**^§^ |  | **Events** | **IR**^§^ | **Events** | **IR**^§^ |  |
| **United States** | | | | | | | | | | |
| **Primary outcome (schizophrenia)** | | | | | | | | | | |
| **Total patients** |  |  |  |  |  |  |  |  |  |  |
| As treated | 2 130 393 | 0.43 | 2 151 531 | 0.37 | 1.11 [0.97-1.28] | 1 777 108 | 0.54 | 1 864 047 | 0.65 | 0.95 [0.83-1.08] |
| Intention-to-treat | 2 134 395 | 0.47 | 2 156 779 | 0.39 | 1.19 [1.14-1.24]* | 1 780 196 | 0.58 | 1 867 321 | 0.64 | 0.92 [0.89-0.95]* |
| **≥45 years** |  |  |  |  |  |  |  |  |  |  |
| As treated | 1 792 503 | 0.41 | 1 813 979 | 0.35 | 1.19 [1.02-1.38]* | 1 316 424 | 0.47 | 1 359 260 | 0.54 | 0.91 [0.77-1.06] |
| Intention-to-treat | 1 797 490 | 0.43 | 1 818 733 | 0.36 | 1.16 [1.11-1.22]* | 1 318 921 | 0.51 | 1 361 765 | 0.54 | 0.96 [0.92-1.00] |
| **Secondary outcome (schizophrenia with ER visit)** | | | | | | | | | | |
| **Total patients** |  |  |  |  |  |  |  |  |  |  |
| As treated | 2 130,393 | 0.02 | 2 151 531 | 0.02 | 1.14 [0.56-2.37] | 1 777 108 | 0.04 | 1 864 047 | 0.04 | 0.88 [0.54-1.43] |
| Intention-to-treat | 2 134 395 | 0.03 | 2 156 779 | 0.02 | 1.27 [1.08-1.50]* | 1 780 196 | 0.05 | 1 867 321 | 0.05 | 0.86 [0.76-0.98]* |
| **≥45 years** |  |  |  |  |  |  |  |  |  |  |
| As treated | 1 792 503 | 0.02 | 1 813 979 | 0.02 | 1.62 [0.82-3.31] | 1 316 424 | 0.02 | 1 359 260 | 0.03 | 0.43 [0.18-0.95]* |
| Intention-to-treat | 1 797 490 | 0.02 | 1 818 733 | 0.02 | 1.22 [1.01-1.49]* | 1 318 921 | 0.03 | 1 361 765 | 0.04 | 0.88 [0.74-1.05] |
| ACE, Angiotensin Converting Enzyme; ARB, Angiotensin II Receptor Blockers, ER, Emergency Room; IR: Incidence rates; HR: hazard ratio; CI: 95% confidence interval;  §Incidence rates were calculated as case per 1 000 person-years;*statistically significant | | | | | | | | | | |

eFigure 3. Risk of secondary outcome events between the ACE inhibitor and the ARB group or between the ACE inhibitor and Thiazide groups

**ACE inhibitor vs ARB**
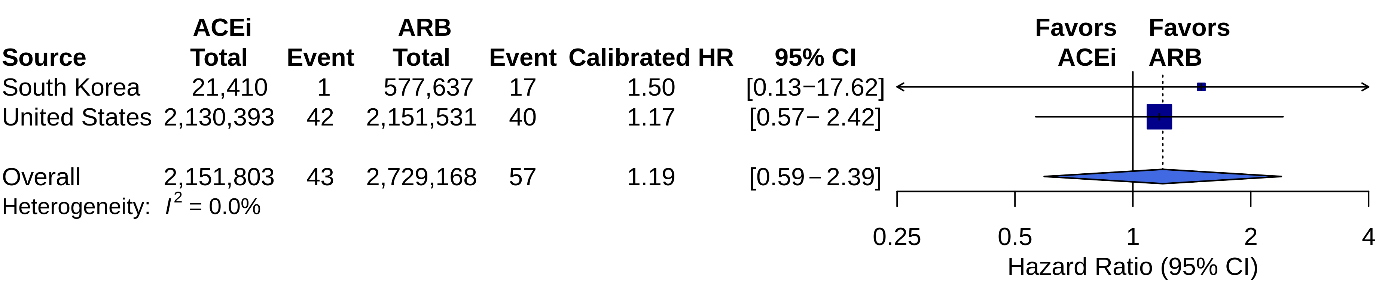


**ACE inhibitor vs thiazide**


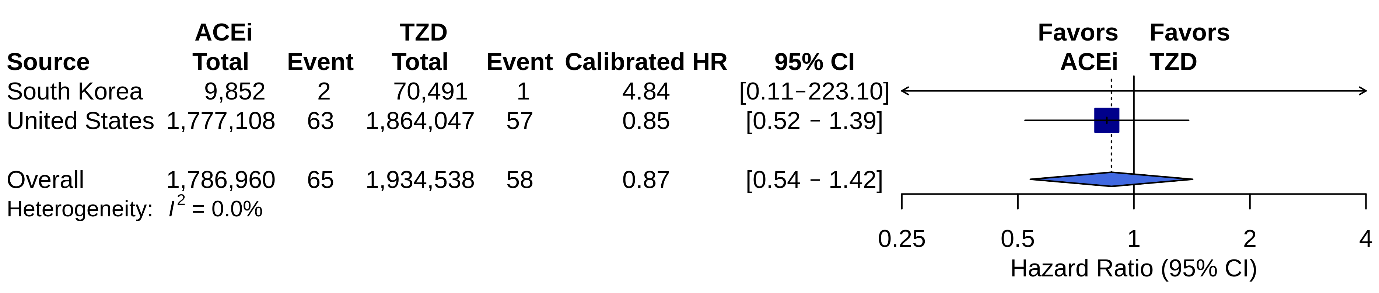


eFigure 4. Risk of primary outcome events between the ACE inhibitor and the ARB group or between the ACE inhibitor and Thiazide groups in the subgroup analysis (Age > 45)

**ACE inhibitor vs ARB**


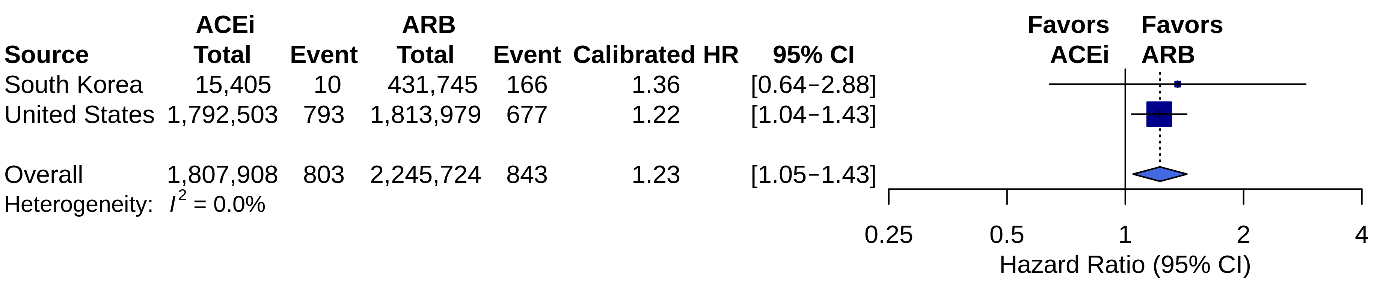


**ACE inhibitor vs thiazide**


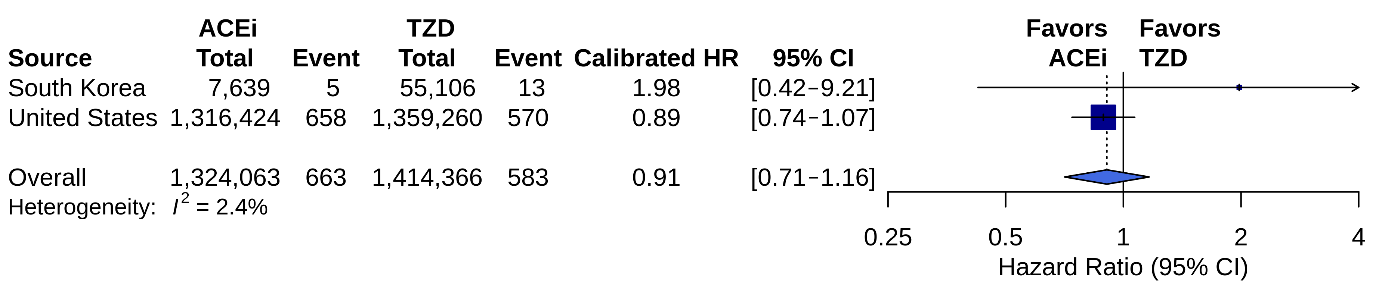


eFigure 5. Risk of secondary outcome events between the ACE inhibitor and the ARB group or between the ACE inhibitor and Thiazide groups in the subgroup analysis (Age > 45)

**ACE inhibitor vs ARB**


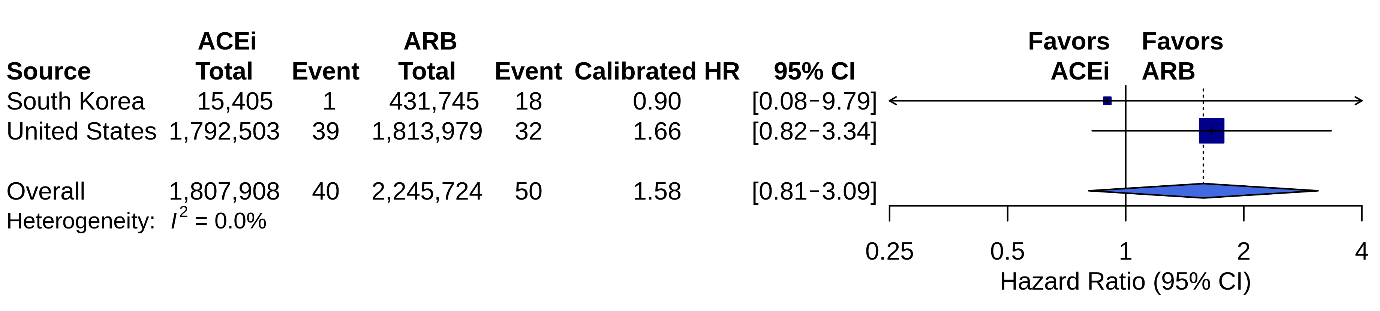


**ACE inhibitor vs thiazide**


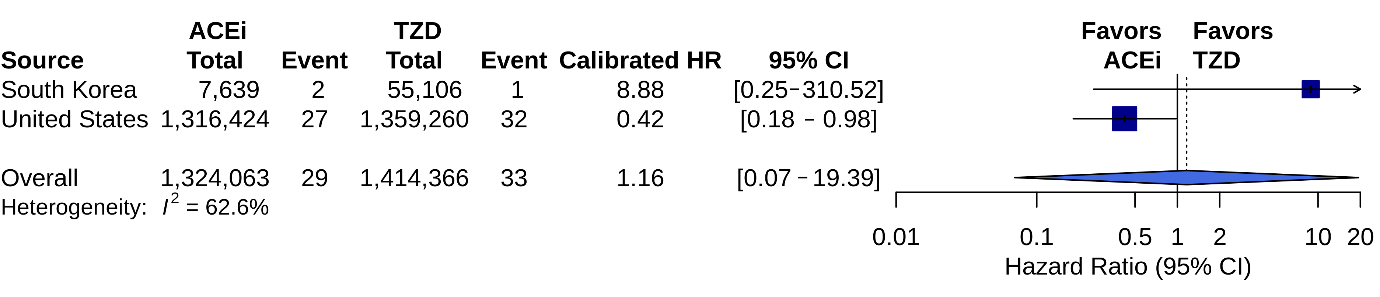


eFigure 6. Risk of primary outcome events between the ACE inhibitor and the ARB group or between the ACE inhibitor and Thiazide groups under ITT

**ACE inhibitor vs ARB**
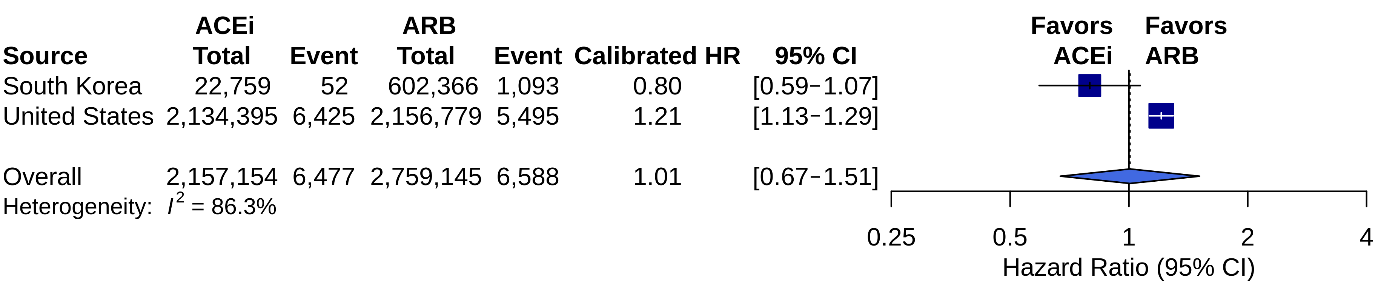


**ACE inhibitor vs thiazide**
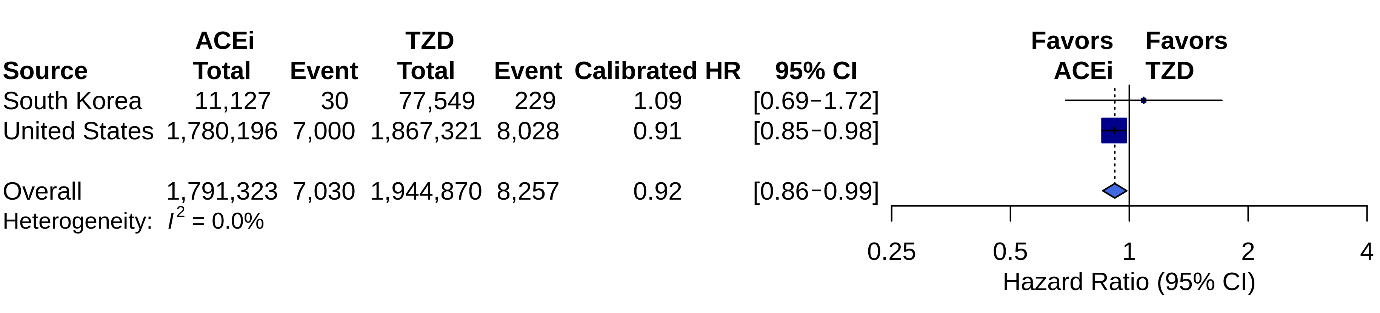


eFigure 7. Risk of primary outcome events between the ACE inhibitor and the ARB group or between the ACE inhibitor and Thiazide groups under ITT in the subgroup analysis (Age > 45)

**ACE inhibitor vs ARB**
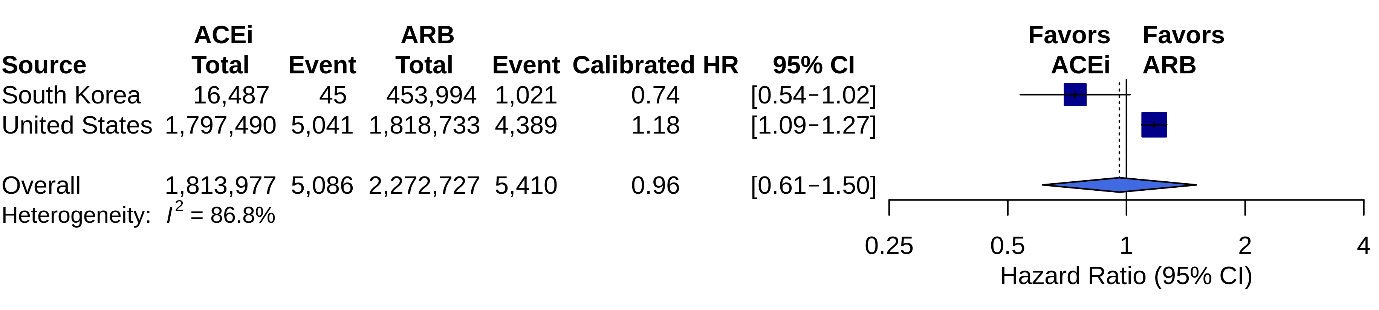


**ACE inhibitor vs thiazide**
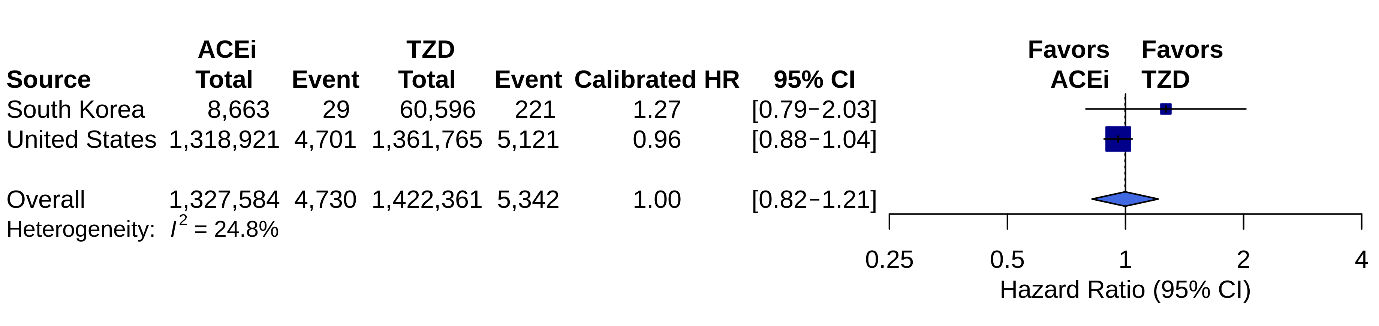


eFigure 8. Risk of secondary outcome events between the ACE inhibitor and the ARB group or between the ACE inhibitor and Thiazide groups under ITT

**ACE inhibitor vs ARB**
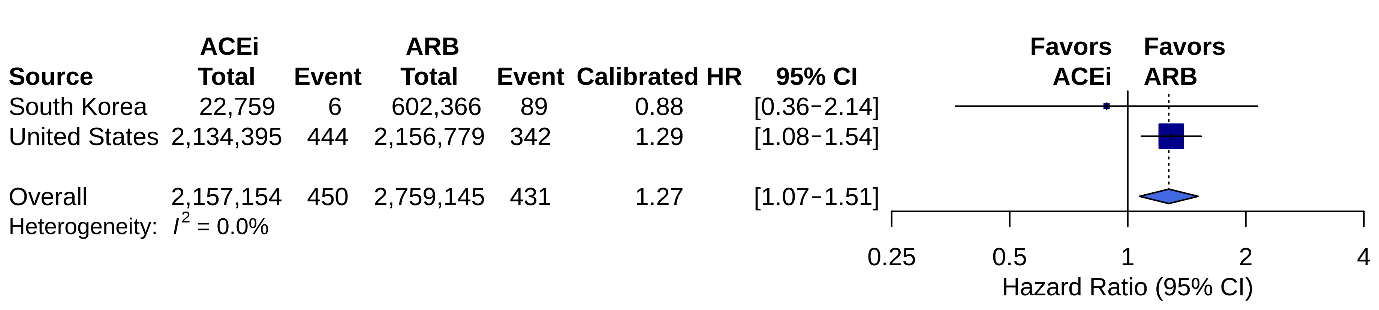


**ACE inhibitor vs thiazide**
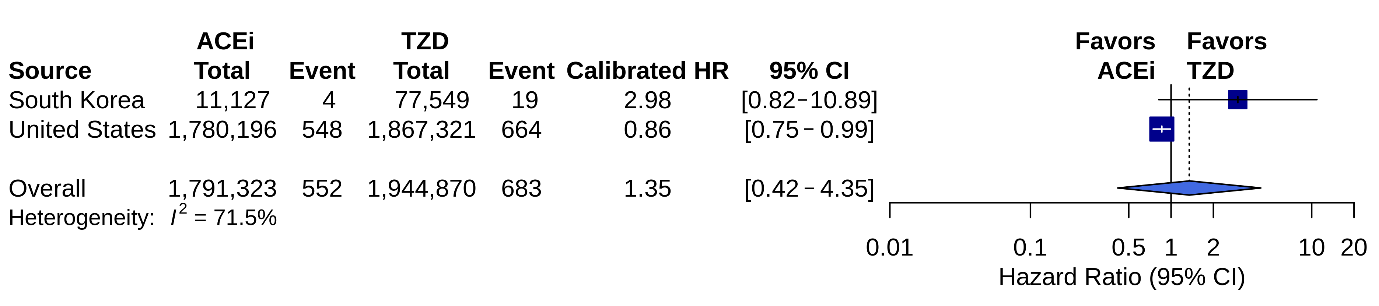


eFigure 9. Risk of secondary outcome events between the ACE inhibitor and the ARB group or between the ACE inhibitor and Thiazide groups under ITT in the subgroup analysis (Age > 45)

**ACE inhibitor vs ARB**
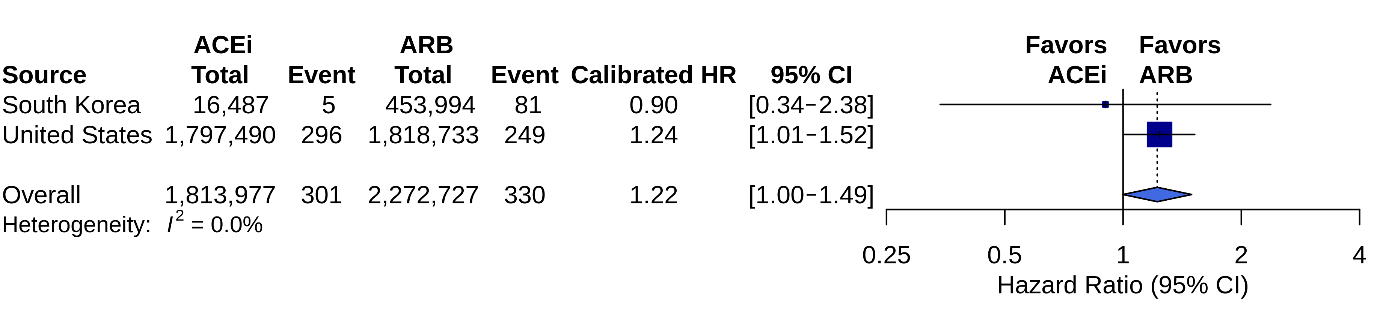


**ACE inhibitor vs thiazide**
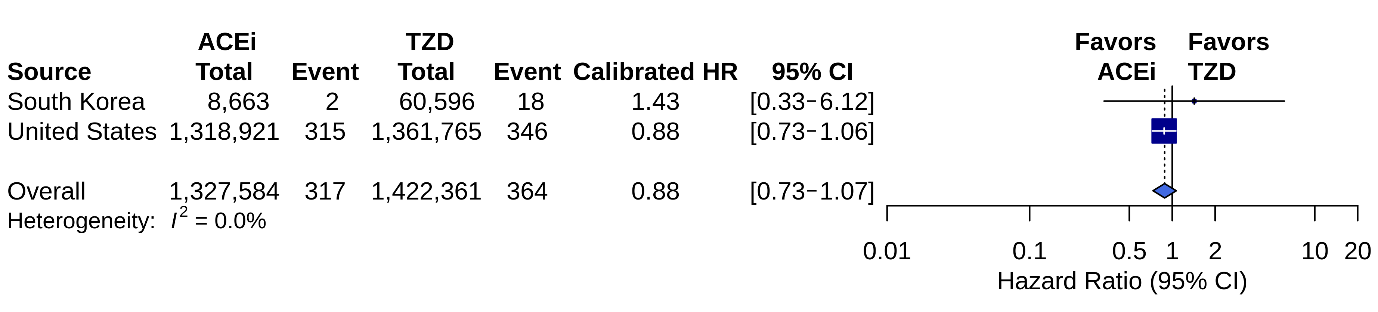

Supplement: Supplementary file 2 — Supplementary Material 2 [file 12888_2024_5578_MOESM2_ESM.docx]
