# Supplementary material for: Comparative estimation of the effects of antihypertensive medications on schizophrenia occurrence: a multinational observational cohort study
Source: BMC Psychiatry. 2024 Feb 16;24:128. doi: 10.1186/s12888-024-05578-6 (PMC10870661; doi:10.1186/s12888-024-05578-6)
Supplement: Supplementary file 3 — Supplementary Material 3 [file 12888_2024_5578_MOESM3_ESM.docx]

**Population-Level Estimation:**

***Comparative Estimation of the Effects of Anti-hypertensive Medications on the Occurrence of Schizophrenia (CEEAMOS)***

**Version:** 1

Dong Yun Lee, MD, Ajou University, Korea

Seng Chan You, MD PhD, Yonsei University, Korea

**Date:** 29 March 2021

**Acknowledgment:** The analysis is based in part on work from the Observational Health Sciences and Informatics collaborative. OHDSI (<http://ohdsi.org>) is a multi-stakeholder, interdisciplinary collaborative to create open-source solutions that bring out the value of observational health data through large-scale analytics.

The authors declare the following disclosures: None

# Table of contents

[1 Table of contents 2](#_Toc68506795)

[2 List of abbreviations 2](#_Toc68506796)

[3 Abstract 3](#_Toc68506797)

[4 Milestones 3](#_Toc68506798)

[5 Rationale and Background 3](#_Toc68506799)

[6 Research Questions and Objectives 4](#_Toc68506800)

[6.1 Research Questions 4](#_Toc68506801)

[6.2 Objectives 5](#_Toc68506802)

[7 Research methods 5](#_Toc68506803)

[7.1 Study Design 5](#_Toc68506804)

[7.2 Data Source(s) 5](#_Toc68506805)

[7.3 Study population 5](#_Toc68506806)

[7.4 Exposures 5](#_Toc68506807)

[7.4.1 All drugs 6](#_Toc68506808)

[7.5 Outcomes 6](#_Toc68506809)

[7.5.1 Schizophrenia 6](#_Toc68506810)

[7.5.2 Schizophrenia diagnosed on first visit to the Emergency Room (ER) 9](#_Toc68506811)

[7.5.3 Negative control outcomes 12](#_Toc68506812)

[7.6 Covariates 13](#_Toc68506813)

[7.6.1 Propensity score covariates 13](#_Toc68506814)

[8 Data Analysis Plan 14](#_Toc68506815)

[8.1 Calculation of time-at risk 14](#_Toc68506816)

[8.2 Model Specification 14](#_Toc68506817)

[8.2.1 Pooling effect estimates across databases 15](#_Toc68506818)

[8.3 Analyses to perform 15](#_Toc68506819)

[8.3.1 Comparative analyses 15](#_Toc68506820)

[8.3.2 Descriptive analyses 15](#_Toc68506821)

[8.4 Output 15](#_Toc68506822)

[8.5 Evidence Evaluation 16](#_Toc68506823)

[9 Study Diagnostics 16](#_Toc68506824)

[9.1 Sample Size and Study Power 16](#_Toc68506825)

[9.2 Cohort Comparability 16](#_Toc68506826)

[9.3 Systematic Error Assessment 16](#_Toc68506827)

[10 Strengths and Limitations of the Research Methods 16](#_Toc68506828)

[11 Protection of Human Subjects 17](#_Toc68506829)

[12 Management and Reporting of Adverse Events and Adverse Reactions 17](#_Toc68506830)

[13 Plans for Disseminating and Communicating Study Results 17](#_Toc68506831)

[14 References 17](#_Toc68506832)

# List of abbreviations

ATC Anatomic Therapeutic Chemical

CYCLOPS Cyclic coordinate descent for logistic, Poisson and survival analysis

SNOMED Systematized Nomenclature of Medicine

OHDSI Observational Health Data Sciences and Informatics

OMOP Observational Medical Outcomes Partnership

T Target cohort

C Comparator cohort

O Outcome cohort

PS Propensity Scores

LASSO Least absolute shrinkage and selection operator

CI Confidence Interval

ECT Electroconvulsive therapy

MedDRA Medical Dictionary for Regulatory Activities

ACE inhibitors Angiotensin Converting Enzyme inhibitors

ARB Angiotensin receptor blocker

CCB Calcium channel blocker

dCCB Dihydropyridine calcium channel blocker

TZD Thiazide

# Abstract

In this study we will generate population-level estimates at scale for one disease: Hypertension. We perform the comparison between hypertension treatments (ACE inhibitors vs Angiotensin receptor blockers (ARBs), ACE inhibitors vs Thiazide (TZD), and ARBs vs TZD) for Schizophrenia occurrence.

Amendments and Updates

| 0.1 | 29 Mar 2021 | DY. Lee, SC. You | Initial draft |
| --- | --- | --- | --- |
| 0.2 | 30 Mar  2021 | DY. Lee, SC. You | Added concept IDs for outcomes of interest |
| 0.3 | 2 Apr  2021 | DY. Lee, SC. You | Added inclusion and exclusion criteria for each study cohort |
| 0.4 | 4 Apr  2021 | DY. Lee, SC. You | Added rationale, background, and associated references |
| 0.5 | 20 Apr  2021 | DY. Lee, SC.  You | Added model specification |
| 0.6 | 7 Jun  2021 | DY. Lee, SC.  You | Added study design |
| 1.0 | 3 Sep  2021 | DY. Lee, SC.  You | Revised study population |

# Milestones

| Milestone | Planned / Estimated Date |
| --- | --- |
| Start of analysis | 29 March 2021 |
| End of analysis |  |
| Presentation of results |  |

# Rationale and Background

Schizophrenia is a mental disorder that affects about 1 percent of the world's population and a severe disorder which leads to functional deterioration.([1](#_ENREF_1)) Schizophrenia usually occurs in late adolescence or early adulthood, and the incidence rate of people aged 45 and under 65 is only 0.6%.([2](#_ENREF_2)) Early detection and intervention of schizophrenia are essential for better prognosis.([3](#_ENREF_3)) Genetic findings have been considered to play important roles in schizophrenia occurrence.([4](#_ENREF_4)) Genome-wide association studies (GWASs) demonstrated more than a hundred susceptibility loci for schizophrenia.([5](#_ENREF_5)) The relative contribution of genetic factors in schizophrenia was estimated at up to 80%.([6](#_ENREF_6)) Some genetic studies have shown an association between antipsychotic response and candidate genes.([7](#_ENREF_7)) However, most of the genetic risk of schizophrenia remains unexplained.([8](#_ENREF_8)) Under these circumstances, finding reliable genetic factors is likely to be important for early diagnosis and treatment.

Recently, it is reported that antihypertensive target gene was associated with schizophrenia risk. Specifically, the results for ACE messenger RNA and protein related to ACE inhibitor were shown.([9](#_ENREF_9)) Observational studies have reported associations between psychiatric disorders and antihypertensive drugs depending on the type of drug.([10](#_ENREF_10)) A recent study has demonstrated the therapeutic potential of angiotensin receptor blockers (ARBs), as adjunctive therapy to antipsychotic medications for schizophrenia treatment. And no effect was observed for thiazide diuretics.([11](#_ENREF_11)) These drugs are all anti-hypertensive drugs, but the effect on schizophrenia seems to be different. Therefore, comparing the effects of antihypertensive drugs on schizophrenia may be a way to find potential factors for early diagnosis and treatment. This study aims to investigate whether ACE inhibitor increases the risk of schizophrenia compared with ARB and thiazide-like diuretics. To look at the association between ACE inhibitor use and incidence of late-onset schizophrenia, a sensitivity analysis will be conducted for older patients. If this study works then, it may provide information on the schizophrenia occurrence influenced by hypertensive treatments. Hence, we aimed to generate population-level estimates across the OHDSI network.

## Research Questions

In this study, we are interested in comparing selected treatments (ACE inhibitors vs Angiotensin receptor blockers (ARBs), ACE inhibitors vs Thiazide (TZD), and ARBs vs TZD) in table 1.

| Drug | Class | Major class |
| --- | --- | --- |
| Benazepril | ACE inhibitors | ACE inhibitors |
| Captopril | ACE inhibitors | ACE inhibitors |
| Enalapril | ACE inhibitors | ACE inhibitors |
| Fosinopril | ACE inhibitors | ACE inhibitors |
| Lisinopril | ACE inhibitors | ACE inhibitors |
| Moexipril | ACE inhibitors | ACE inhibitors |
| Perindopril | ACE inhibitors | ACE inhibitors |
| Quinapril | ACE inhibitors | ACE inhibitors |
| Ramipril | ACE inhibitors | ACE inhibitors |
| Trandolapril | ACE inhibitors | ACE inhibitors |
| Azilsartan | Angiotensin receptor blockers (ARBs) | Angiotensin receptor blockers (ARBs) |
| Candesartan | Angiotensin receptor blockers (ARBs) | Angiotensin receptor blockers (ARBs) |
| Eprosartan | Angiotensin receptor blockers (ARBs) | Angiotensin receptor blockers (ARBs) |
| irbesartan | Angiotensin receptor blockers (ARBs) | Angiotensin receptor blockers (ARBs) |
| Losartan | Angiotensin receptor blockers (ARBs) | Angiotensin receptor blockers (ARBs) |
| Olmesartan | Angiotensin receptor blockers (ARBs) | Angiotensin receptor blockers (ARBs) |
| Telmisartan | Angiotensin receptor blockers (ARBs) | Angiotensin receptor blockers (ARBs) |
| Valsartan | Angiotensin receptor blockers (ARBs) | Angiotensin receptor blockers (ARBs) |
| Chlorthalidone | Thiazide or thiazide-like diuretics | Diuretics |
| Hydrochlorothiazide | Thiazide or thiazide-like diuretics | Diuretics |
| Indapamide | Thiazide or thiazide-like diuretics | Diuretics |
| Metolazone | Thiazide or thiazide-like diuretics | Diuretics |

**Table 1**. List of hypertension treatments considered in this study

For comparing the two treatments, we are interested in the comparative effect on outcomes listed in table 2.

| Schizophrenia | Schizophrenia diagnosed on first visit to the Emergency Room (ER) |
| --- | --- |

**Table 2.** The outcome in this study

Research question

- For comparing the two hypertension treatments, for the schizophrenia occurrence, what is the hazard ratio?
- What are the results in older populations compared to the total population?

## Objectives

Objective

- Generate evidence for the comparative effects for selected treatments (ACE inhibitors vs Angiotensin receptor blockers (ARBs), ACE inhibitors vs Thiazide (TZD), and ARBs vs TZD) for the outcome of interest.

# Research methods

## Study Design

This study will be a set of retrospective, observational, new-user cohort studies. By ‘retrospective’ we mean the study will use data already collected at the start of the study. By ‘observational’ we mean no intervention will take place in the course of this study. By ‘new-user’ we mean we will only analyze the first exposure of a subject to the treatment of interest. By ‘cohort study’ we mean two cohorts, a target and comparator cohort, will be followed from index date (start of first exposure) to some end date, and assessed for the occurrence of the outcomes of interest.

## Data Source(s)

## Study population

All subjects in the database will be included who meet the following criteria: (note: the index date is the start of the first treatment for hypertension)

- Exposure to one of the treatments of interest
- At least 365 days of observation time prior to the index date
- No exposure of any hypertension treatment before the index date
- A diagnose of hypertensive disorder on or preceding the index date
- No diagnose of the outcome of interest preceding the index date
- No diagnose of heart failure preceding the index date
- With age greater or equal to 18

## Exposures

In this study, we are interested in the comparison between two treatments in table 1.

### All drugs

Index rule defining the index date:

- First exposure to ACE inhibitors or ARBs or TZD.

Inclusion rules based on the index date:

- At least 365 days of observation time prior to the index date
- No exposure to the target or comparator ingredient(s) before the index date
- No exposure to any other hypertension treatment on or before the index date
- No diagnose of heart failure preceding the index date
- A diagnosis of hypertension within 365 days before the index date starts

Note that no prior exposure to other hypertension treatments prior to or on the index date is allowed. For example, when comparing ACE inihibitor to ARB, no prior exposure to thiazide or any other hypertension treatment is allowed.

The end of the exposure cohort is defined as the end of the first exposure, allowing for 30-day gaps between consecutive prescriptions.

## Outcomes

### Schizophrenia

Persons with schizophrenia

The first condition record of schizophrenia, which is followed by another schizophrenia condition record, at least two drugs used to treat schizophrenia without another indication, or two psychotherapy procedures

Initial Event Cohort

People having any of the following: 

- a condition occurrence of Schizophrenia
  - for the first time in the person's history

with continuous observation of at least 0 days prior and 0 days after event index date, and limit initial events to: **earliest event per person.**

For people matching the Primary Events, include:

Having any of the following criteria:

- at least 1 occurrences of a condition occurrence of Schizophrenia

where event starts between 1 days After and all days After index start date

- or at least 2 occurrences of a drug exposure of Drugs to treat Schizophrenia

Having all of the following criteria:

- - exactly 0 occurrences of a condition occurrence of Other indications for drugs used to treat schizophrenia

where event starts between 30 days Before and 7 days After index start date

where event starts between 0 days Before and all days After index start date

- or at least 2 occurrences of a procedure of Procedures for schizophrenia

where event starts between 0 days Before and all days After index start date

Limit cohort of initial events to: **earliest event per person.**

Limit qualifying cohort to: **earliest event per person.**

End Date Strategy

The cohort event will persist until fixed duration relative to initial event.

- Event date to offset from: start date
- Number of days offset: 0 days

Cohort Collapse Strategy:

Collapse cohort by era with a gap size of 0 days.

Appendix 1: Concept Set Definitions

1. Schizophrenia

| Concept Id | Concept Name | Domain | Vocabulary | Excluded | Descendants | Mapped |
| --- | --- | --- | --- | --- | --- | --- |
| 435783 | Schizophrenia | Condition | SNOMED | NO | YES | NO |

2. Drugs to treat schizophrenia

| Concept Id | Concept Name | Domain | Vocabulary | Excluded | Descendants | Mapped |
| --- | --- | --- | --- | --- | --- | --- |
| 21604490 | ANTIPSYCHOTICS | Drug | ATC | NO | YES | NO |

3. Other indications for drugs used to treat schizophrenia

| Concept Id | Concept Name | Domain | Vocabulary | Excluded | Descendants | Mapped |
| --- | --- | --- | --- | --- | --- | --- |
| 436665 | Bipolar disorder | Condition | SNOMED | NO | YES | NO |
| 440383 | Depressive disorder | Condition | SNOMED | NO | YES | NO |
| 4182210 | Dementia | Condition | SNOMED | NO | YES | NO |
| 4286201 | Schizoaffective disorder | Condition | SNOMED | NO | YES | NO |

4. Procedures for schizophrenia

| Concept Id | | Concept Name | | Domain | Vocabulary | | | Excluded | | Descendants | | Mapped |
| --- | --- | --- | --- | --- | --- | --- | --- | --- | --- | --- | --- | --- |
| 4030840 | Electroconvulsive therapy | | Procedure | | | SNOMED | NO | | YES | | NO | |
| 2795842 | Mental Health, Electroconvulsive Therapy | | Procedure | | | ICD10PCS | NO | | YES | | NO | |
| 2795675 | Mental Health, Individual Psychotherapy | | Procedure | | | ICD10PCS | NO | | YES | | NO | |
| 4327941 | Psychotherapy | | Procedure | | | SNOMED | NO | | YES | | NO | |
| 45887951 | Psychotherapy Services and Procedures | | Procedure | | | CPT4 | NO | | YES | | NO | |

### Schizophrenia diagnosed on first visit to the Emergency Room (ER)

Persons with schizophrenia diagnosed on first visit to the Emergency Room (ER)

The first condition record of schizophrenia, which is followed by another schizophrenia condition record, at least two drugs used to treat schizophrenia without another indication, or two psychotherapy procedures

Initial Event Cohort

People having any of the following: 

- a condition occurrence of Schizophrenia
  - for the first time in the person's history

Having all of the following criteria

- - - a visit occurrence of ER visit

where event starts between 0 days before and 0days after index start date

restrict to the same visit occurrence

with continuous observation of at least 0 days prior and 0 days after event index date, and limit initial events to: **earliest event per person.**

For people matching the Primary Events, include:

Having any of the following criteria:

- at least 1 occurrences of a condition occurrence of Schizophrenia

where event starts between 1 days After and all days After index start date

- or at least 2 occurrences of a drug exposure of Drugs to treat Schizophrenia

Having all of the following criteria:

- - exactly 0 occurrences of a condition occurrence of Other indications for drugs used to treat schizophrenia

where event starts between 30 days Before and 7 days After index start date

where event starts between 0 days Before and all days After index start date

- or at least 2 occurrences of a procedure of Procedures for schizophrenia

where event starts between 0 days Before and all days After index start date

Limit cohort of initial events to: **earliest event per person.**

Limit qualifying cohort to: **earliest event per person.**

End Date Strategy

The cohort event will persist until fixed duration relative to initial event.

- Event date to offset from: start date
- Number of days offset: 0 days

Cohort Collapse Strategy:

Collapse cohort by era with a gap size of 0 days.

Appendix 1: Concept Set Definitions

1. Schizophrenia

| Concept Id | Concept Name | Domain | Vocabulary | Excluded | Descendants | Mapped |
| --- | --- | --- | --- | --- | --- | --- |
| 435783 | Schizophrenia | Condition | SNOMED | NO | YES | NO |

2. ER visit

| Concept Id | Concept Name | Domain | Vocabulary | Excluded | Descendants | Mapped |
| --- | --- | --- | --- | --- | --- | --- |
| 262 | Emergency Room and Inpatient Visit | Visit | Visit | NO | YES | NO |
| 9203 | Emergency Room Visit | Visit | Visit | NO | YES | NO |

3. Drugs to treat schizophrenia

| Concept Id | Concept Name | Domain | Vocabulary | Excluded | Descendants | Mapped |
| --- | --- | --- | --- | --- | --- | --- |
| 21604490 | ANTIPSYCHOTICS | Drug | ATC | NO | YES | NO |

4. Other indications for drugs used to treat schizophrenia

| Concept Id | Concept Name | Domain | Vocabulary | Excluded | Descendants | Mapped |
| --- | --- | --- | --- | --- | --- | --- |
| 436665 | Bipolar disorder | Condition | SNOMED | NO | YES | NO |
| 440383 | Depressive disorder | Condition | SNOMED | NO | YES | NO |
| 4182210 | Dementia | Condition | SNOMED | NO | YES | NO |
| 4286201 | Schizoaffective disorder | Condition | SNOMED | NO | YES | NO |

5. Procedures for schizophrenia

| Concept Id | | Concept Name | | Domain | Vocabulary | | | Excluded | | Descendants | | Mapped |
| --- | --- | --- | --- | --- | --- | --- | --- | --- | --- | --- | --- | --- |
| 4030840 | Electroconvulsive therapy | | Procedure | | | SNOMED | NO | | YES | | NO | |
| 2795842 | Mental Health, Electroconvulsive Therapy | | Procedure | | | ICD10PCS | NO | | YES | | NO | |
| 2795675 | Mental Health, Individual Psychotherapy | | Procedure | | | ICD10PCS | NO | | YES | | NO | |
| 4327941 | Psychotherapy | | Procedure | | | SNOMED | NO | | YES | | NO | |
| 45887951 | Psychotherapy Services and Procedures | | Procedure | | | CPT4 | NO | | YES | | NO | |

### Negative control outcomes

Negative controls are concepts known to not be associated with the target or comparator cohorts, such that we can assume the true relative risk between the two cohorts is 1. Negative controls are selected using a similar process to that outlined by Voss et al. Person counts of all potential drug-condition pairs are reviewed in observational data; this person count data helps determine which pairs are even probable for use in calibration. Given the list of potential drug-condition pairs, the concepts in the pairs must meet the following requirements to be considered as negative controls: (1) that there is no Medline abstract where the MeSH terms suggest an association between the drug and the condition, (2) that there is no mention of the drug-condition pair on a US Product Label in the “Adverse Drug Reactions” or “Postmarketing” section, (3) there are no US spontaneous reports suggesting that the pair is in an adverse event relationship, (4) that the OMOP Vocabulary does not suggest that the drug is indicated for the condition, (5) that the concepts are usable (i.e. not too broad, not suggestive of an adverse event relationship, not pregnancy related), and (6) the exact concept itself is utilized in patient level data (i.e. concepts that are not usually used within the data are usually indicative a broad concept that has a child that is more specific). The remaining concepts are “optimized”, meaning parent concepts remove children as defined by the OMOP Vocabulary (e.g. if both “Non-Hodgkin’s Lymphoma” and “B-Cell Lymphoma” we selected, child concept “B-Cell Lymphoma would be removed for its parent “Non-Hodgkin’s Lymphoma”). Once potential negative control candidates were selected, manual clinical review to exclude any pairs that may still be in a causal relationship or similar to the study outcome was be performed to select the top concepts by patient exposure. The final list can be found in Table 3.

| Abnormal cervical smear | Homocystinuria |
| --- | --- |
| Abnormal pupil | Human papilloma virus infection |
| Abrasion and/or friction burn of trunk without infection | Ileostomy present |
| Absence of breast | Impacted cerumen |
| Absent kidney | Impingement syndrome of shoulder region |
| Acid reflux | Ingrowing nail |
| Acquired hallux valgus | Injury of knee |
| Acquired keratoderma | Irregular periods |
| Acquired trigger finger | Kwashiorkor |
| Acute conjunctivitis | Late effect of contusion |
| Amputated foot | Late effect of motor vehicle accident |
| Anal and rectal polyp | Leukorrhea |
| Burn of forearm | Macular drusen |
| Calcaneal spur | Melena |
| Cannabis abuse | Nicotine dependence |
| Cervical somatic dysfunction | Noise effects on inner ear |
| Changes in skin texture | Nonspecific tuberculin test reaction |
| Chondromalacia of patella | Non-toxic multinodular goiter |
| Cocaine abuse | Onychomycosis due to dermatophyte |
| Colostomy present | Opioid abuse |
| Complication due to Crohn's disease | Passing flatus |
| Contact dermatitis | Postviral fatigue syndrome |
| Contusion of knee | Presbyopia |
| Crohn's disease | Problem related to lifestyle |
| Derangement of knee | Psychalgia |
| Difficulty sleeping | Ptotic breast |
| Disproportion of reconstructed breast | Regular astigmatism |
| Effects of hunger | Senile hyperkeratosis |
| Endometriosis | Somatic dysfunction of lumbar region |
| Epidermoid cyst | Splinter of face, without major open wound |
| Feces contents abnormal | Sprain of ankle |
| Foreign body in orifice | Strain of rotator cuff capsule |
| Ganglion cyst | Tear film insufficiency |
| Genetic predisposition | Tobacco dependence syndrome |
| Hammer toe | Vaginitis and vulvovaginitis |
| Hereditary thrombophilia | Verruca vulgaris |
| Herpes zoster without complication | Wrist joint pain |
| High risk sexual behavior | Wristdrop |

**Table 3**. Negative control outcomes

For each negative control outcome, a patient enters the negative control outcome cohort at the occurrence of a diagnose code identified by the concepts listed above, or any one of its descendant codes.

## Covariates

### Propensity score covariates

Propensity scores (PS) will be used as an analytic strategy to reduce potential confounding due to imbalance between the target and comparator cohorts in baseline covariates. The propensity score is the probability of a patient being classified in the target cohort vs. the comparator cohort, given a set of observed covariates.

The types of baseline covariates used to fit the propensity score model will be:

- Demographics
  - Gender
  - Age
  - Age group (5-year bands)
  - Index year
  - Index month
- Conditions
  - In prior 30d
  - In prior 365d
- Condition aggregation
  - SNOMED
- Drugs
  - In prior 30d
  - In prior 365d
  - Overlapping index date
- Drug aggregation
  - Ingredient
  - ATC Class
- Procedure
  - In prior 30d
  - In prior 365d
- Risk scores
  - Charlson comorbidity index

All covariates that occur in fewer than 0.1% of the persons between the target and comparator cohorts combined will be excluded prior to model fitting for computational efficiency.

# Data Analysis Plan

## Calculation of time-at risk

Two time-at-risk periods will be used:

- On-treatment. Starting on the day of treatment initiation, and stopping at treatment end, allowing for a maximum gap of 30 days between prescriptions.
- Intent-to-treat: Starting on the day of treatment initiation and stopping at the end of observation.

## Model Specification

In this study, we compare the target cohort with the comparator cohort for the hazards of outcome during the time-at-risk by applying a Cox proportional hazards model.

The time-to-event of outcome among patients in the target and comparator cohorts is determined by calculating the number of days from the start of the time-at-risk window (the cohort start date), until the earliest event among 1) the first occurrence of the outcome, 2) the end of the time-at-risk window as defined in section 9.1 (i.e. ‘on-treatment’ or ‘intent-to-treat’), and 3) the end of the observation period that spans the time-at-risk start.

Patients with the outcome observed prior to target or comparator cohort entry are excluded from consideration.

Propensity scores will be used as an analytic strategy to reduce potential confounding due to imbalance between the target and comparator cohorts in baseline covariates. The propensity score is the probability of a patient being classified in the target cohort vs. the comparator cohort, given a set of observed covariates. In this study, the propensity score is estimated for each patient, using the predicted probability from a regularized logistic regression model, fit with a Laplace prior (LASSO) and the regularization hyperparameter selected by optimizing the likelihood in a 10-fold cross, a starting variance of 0.01 and a tolerance of 2e-7. Covariates to be used in the propensity score model are listed in section 8.6.

In one analysis the target cohort and comparator cohorts will be stratified into ten quantiles of the propensity score distribution. A second analysis will use variable ratio matching based on the propensity score, using a caliper of 0.2 on the standardized logit scale. The final outcome model will apply a conditional Cox proportional hazard model, conditioned on the propensity score strata or matched sets.

Incidence rates will be computed for each outcome in each exposure group, in both the on-treatment and intent-to-treat windows.

### Pooling effect estimates across databases

Effects will be pooled across databases using a random-effects meta-analysis. Estimates for negative and positive controls will be pooled before performing empirical calibration on the pooled estimates.

## Analyses to perform

### Comparative analyses

The following comparative analyses will be performed if sufficient data is present

- 72 comparison between two anti-hypertensive treatments
  - Target class*Outcome of interest*Analysis settings (3*2*12) =72
- 2 outcomes of interest
- 2 age definitions: All adults (greater or equal to 18) and older adults (greater than 45)
- 2 time-at-risk definitions: on-treatment and intent-to-treat
- 3 models: Cox regression using propensity score stratification and Cox regression using 1:1 and variable ratio propensity score matching

### Descriptive analyses

The following incidence rate computations will be performed:

- three cohorts of interest:
  - ACE inhibitor
  - ARBs
  - TZD
- 2 outcomes of interest
- 2 time-at-risk definitions: on-treatment and intent-to-treat
- databases:

## Output

The output will be stored, which is described elsewhere.

## Evidence Evaluation

We have executed diagnostics to determine if the analysis can be appropriately conducted. The diagnostics include:

- Propensity score distribution
- Covariate balance before and after propensity score matching
- Estimation for negative controls, to assess residual error
- Negative control exposures and outcomes will be used to evaluate the potential impact of residual systematic error in the study design, and to facilitate empirical calibration of the p-value and confidence interval for the exposures and outcome of interest.

Negative control outcomes in the context of this study are outcomes that are not believed to be caused by neither exposure in any comparison and where therefore the true hazard ratio is equal to 1. We will execute the same analysis used for the primary hypotheses to produce hazard ratio estimates for the negative controls. The distribution of effect estimates across all negative controls will be used to fit an empirical null distribution which models the observed residual systematic error. The empirical null distribution will then be applied to the target exposures and outcome of interest to calibrate the p-value.

Empirical calibration serves as an important diagnostic tool to evaluate if the residual systematic error is sufficient to cast doubt on the accuracy of the unknown effect estimate. The calibration effect plot and calibration probability plots will be generated for review. We will report the traditional and empirically calibrated p-value and confidence interval for each negative control, as well as the hypothesis of interest.

# Study Diagnostics

## Sample Size and Study Power

This will be reported in the output.

## Cohort Comparability

This will be reported in the output.

## Systematic Error Assessment

This will be reported in the output.

# Strengths and Limitations of the Research Methods

Strength

- Cohort studies allow direct estimation of incidence rates following exposure of interest, and the new-user design can capture early events following treatment exposures while avoiding confounding from previous treatment effects. New use allows for a clear exposure index date.
- PS matching allow balancing on a large number of baseline potential confounders.
- Use of negative and positive control outcomes allow for evaluating the study design as a whole in terms of residual bias.

Limitations

- Even though many potential confounders will be included in this study, there may be residual bias due to unmeasured or misspecified confounders.

# Protection of Human Subjects

The study is using only de-identified data. Confidentiality of patient records will be maintained at all times. All study reports will contain aggregate data only and will not identify individual patients or physicians.

# Management and Reporting of Adverse Events and Adverse Reactions

This study uses coded data that already exist in an electronic database. In this type of database, it is not possible to link (i.e., identify a potential causal association between) a particular product and medical event for any individual. Thus, the minimum criteria for reporting an adverse event (i.e., identifiable patient, identifiable reporter, a suspect product, and event) are not available and adverse events are not reportable as individual adverse events reports. The study results will be assessed for medically important results.

# Plans for Disseminating and Communicating Study Results

The study results will be posted on the OHDSI website after completion of the study. At least one paper describing the study and its results will be written and submitted for publication to a peer-reviewed scientific journal.

# References

1. Jablensky A, Sartorius N, Ernberg G, Anker M, Korten A, Cooper JE, et al. Schizophrenia: manifestations, incidence and course in different cultures A World Health Organization Ten-Country Study. Psychological Medicine Monograph Supplement. 1992;20:1-97.

2. Howard R, Rabins PV, Seeman MV, Jeste DV, Late-Onset tI. Late-onset schizophrenia and very-late-onset schizophrenia-like psychosis: an international consensus. American Journal of Psychiatry. 2000;157(2):172-8.

3. Lin C-H, Lane H-Y. Early identification and intervention of schizophrenia: insight from hypotheses of glutamate dysfunction and oxidative stress. Frontiers in psychiatry. 2019;10:93.

4. Birnbaum R, Weinberger DR. Genetic insights into the neurodevelopmental origins of schizophrenia. Nature Reviews Neuroscience. 2017;18(12):727-40.

5. Ikeda M, Takahashi A, Kamatani Y, Momozawa Y, Saito T, Kondo K, et al. Genome-wide association study detected novel susceptibility genes for schizophrenia and shared trans-populations/diseases genetic effect. Schizophrenia bulletin. 2019;45(4):824-34.

6. Giegling I, Hosak L, Mössner R, Serretti A, Bellivier F, Claes S, et al. Genetics of schizophrenia: A consensus paper of the WFSBP Task Force on Genetics. The world journal of biological psychiatry. 2017;18(7):492-505.

7. Vita A, Minelli A, Barlati S, Deste G, Giacopuzzi E, Valsecchi P, et al. Treatment-resistant schizophrenia: genetic and neuroimaging correlates. Frontiers in pharmacology. 2019;10:402.

8. Greenwood TA, Lazzeroni LC, Maihofer AX, Swerdlow NR, Calkins ME, Freedman R, et al. Genome-wide association of endophenotypes for schizophrenia from the Consortium on the Genetics of Schizophrenia (COGS) study. JAMA psychiatry. 2019;76(12):1274-84.

9. Chauquet S, Zhu Z, O’Donovan MC, Walters JT, Wray NR, Shah S. Association of Antihypertensive Drug Target Genes With Psychiatric Disorders: A Mendelian Randomization Study. JAMA psychiatry. 2021.

10. Hayes JF, Lundin A, Wicks S, Lewis G, Wong IC, Osborn DP, et al. Association of hydroxylmethyl glutaryl coenzyme A reductase inhibitors, L-Type calcium channel antagonists, and biguanides with rates of psychiatric hospitalization and self-harm in individuals with serious mental illness. JAMA psychiatry. 2019;76(4):382-90.

11. Lintunen J, Lähteenvuo M, Tiihonen J, Tanskanen A, Taipale H. Adenosine modulators and calcium channel blockers as add-on treatment for schizophrenia. NPJ schizophrenia. 2021;7(1):1-7.
